# Two-season agriculture and irrigated rice during the Dian: radiocarbon dates and archaeobotanical remains from Dayingzhuang, Yunnan, Southwest China

**DOI:** 10.1007/s12520-020-01268-y

**Published:** 2021-03-13

**Authors:** Rita Dal Martello, Xiaorui Li, Dorian Q. Fuller

**Affiliations:** 1 Department of Archaeology, Max Planck Institute for the Science of Human History, Kahlaische Strasse 10, 07745 Jena, Germany; 2 Yunnan Province Institute of Cultural Relics and Archaeology, Kunming, 650118 China; 3 Institute of Archaeology, University College London, 31-34 Gordon Square, London, WC1H 0PY UK; 4 School of Cultural Heritage, Northwest University, Xi’an, 710069 China

**Keywords:** Bronze Age, Archaeobotany, Oryza, Millet, Wheat, Southwestern barbarians

## Abstract

Historical sources describe irrigation and intensive agriculture being practiced in lowland Yunnan from at least the first century AD, but so far archaeobotanical remains allowing investigation of this issue have been scarce. Here, we present new archaeobotanical evidence, including macro-botanical and phytoliths results, from the Dian settlement site of Dayingzhuang, with direct AMS radiocarbon dates on two wheat grains falling between 750 and 390 BC. We compare these results with contemporary Dian sites and analyse the agricultural systems in Central Yunnan between the eight and fourth centuries BC. We propose that agriculture was intensified toward the end of the Dian through both multiple cropping seasons and increased evidence for irrigated rice fields.

**Supplementary Information:**

The online version contains supplementary material available at 10.1007/s12520-020-01268-y.

## Introduction

The subsistence of people living in Yunnan prior and during the Dian Culture has been a topic of recent interest (i.e. Dal Martello 2020; Wu et al. 2019; Yao 2016; YPICRA et al. 2015; Yao and Jiang 2012). Formerly known as “Shizhaishan Culture”, archaeological evidence now associated with the Dian was first discovered in 1955 during the excavation of the burial site of the same name. Shizhaishan cemetery is located on the Southwestern bank of the Lake Dian; sophisticated bronze objects were found in the graves, including weapons, drum-shaped cowrie shell containers, and most notably a gold seal bearing the inscription: “The Seal of the King of Dian” (see Yao 2017; Yao and Jiang 2012; Yunnan 1963). This led to the hypothesis that the Dian Culture was part of the “Southwestern Barbarians” as referred to in early Chinese historical texts (see for example the *Shiji*, 史记 *Records of a Grand Historian*; Shiji 116; Qian 1993; Yunnan 1963). According to current archaeological evidence, the Dian were present in Yunnan from at least the eighth century BC, until they were conquered by the Han Dynasty in 109 BC (Zhang 1997; Allard 1999).

Direct archaeobotanical evidence from Dian sites has only recently began to become available, and most previous theories on Dian subsistence practices drew from historical records, written by Han historians following 109 BC. These describe the presence of irrigated rice fields in Yunnan’s lowlands from at least 16 AD (Yao et al. 2015). Funerary clay figurines of rice paddy fields have been recovered from first century AD Han-style graves in Dali, Dian and Gejiu (Yao 2016). These are usually interpreted as a reflection of the deceased possessions and lifestyle before death, and would, therefore, further attest to the presence of irrigated rice cultivation in Yunnan from at least the first century AD. Paleo-environmental studies on Lake Dian sediments indicate abrupt changes in the palynological records from the fifth century BC; this has been interpreted as a possible indication of the beginning of irrigation and terracing activities (Sun et al. 1986; Xiao et al. 2020). However, others have instead argued against such an early date for irrigation in Central Yunnan, with lake sediments from the nearby Lake Xingyun, located about 70 km southeast from the Lake Dian, showing minimal impact on lake water management before 500 AD (Hillman et al. 2014). The Lake Dian is surrounded by alluvial plains which would have provided suitable soil for intensive agricultural production through irrigation; however, conclusive evidence for when exactly irrigation practices and intensification of agriculture started in Yunnan is still lacking.

**Fig. 1:**
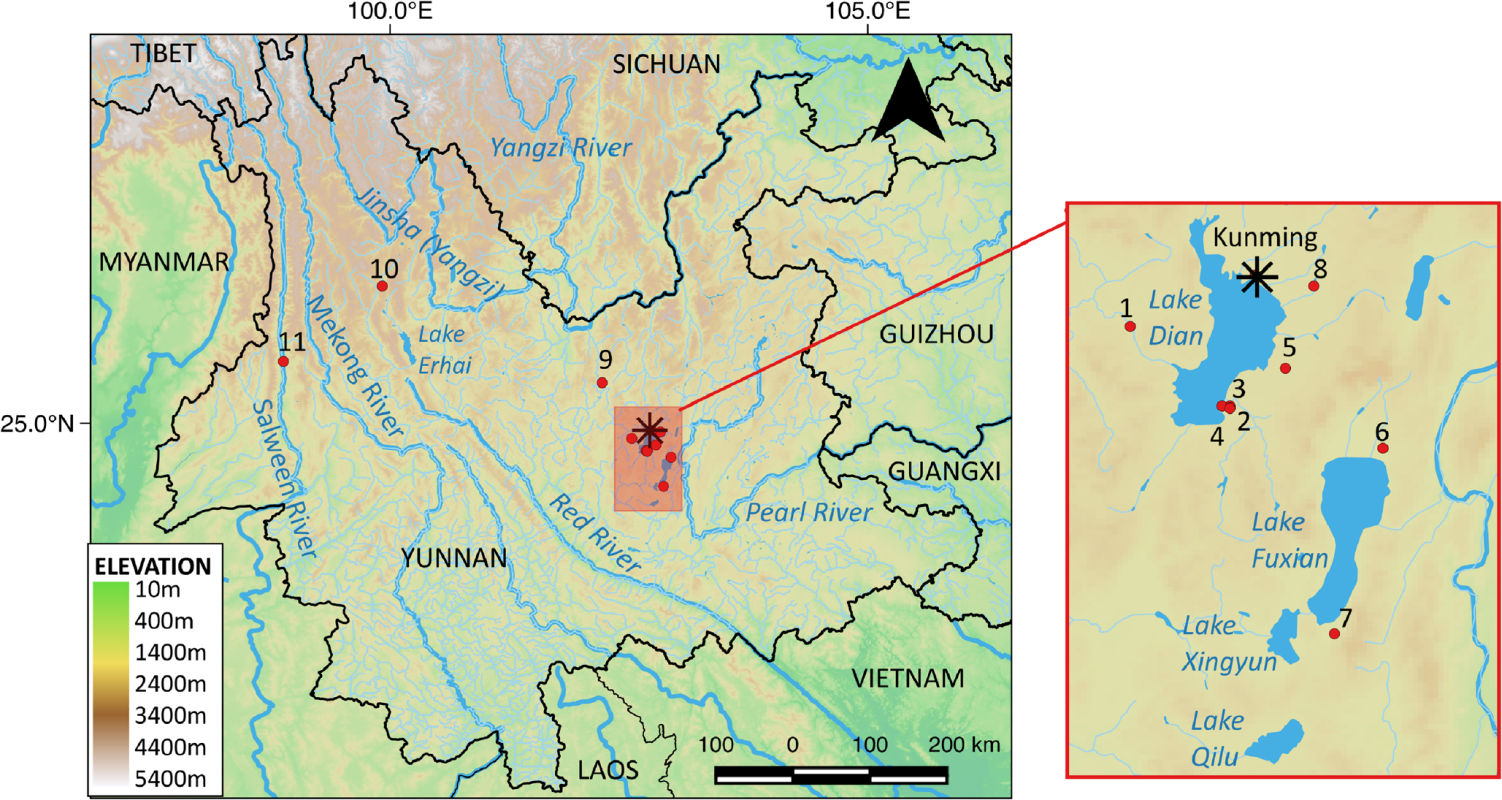
Map showing location of Bronze Age sites in Yunnan mentioned in text: 1. Dayingzhuang; 2. Shizhaishan; 3. Hebosuo; 4. Shangxihe; 5. Anjiang; 6. Xueshan; 7. Guangfentou; 8. Xiaogucheng; 9. Yubeidi; 10. Haimenkou; 11. Shilinggang. Made with QGIS 3.10.10

In this paper, we present archaeobotanical evidence and direct radiocarbon dates from the recently excavated site of Dayingzhuang, a Dian settlement site situated less than 40 km away from the modern capital city of Kunming (Fig. 1). The site sits at the mouth of the Tanglang River, which flows into the Lake Dian and connects its western bank to the Anning area, a centre for the production of copper during the Dian (YPICRA et al. 2015). There is an excavated cemetery in this area (YPAT 1965) and additional contemporary settlement sites along the upper and lower Tanglang Basin that attest to contacts between the broader Dian Basin with the Jinsha Basin, in the northwest of the province (YPICRA et al. 2015). Foxtail millet (*Setaria italica*) was reported from flotation samples collected from an exposed pit profile at Dayingzhuang during a survey in 2010 (YPICRA et al. 2015: 158); however, no formal archaeobotanical report was published. Here, we present for the first time archaeobotanical results from 30 flotation and 11 phytolith samples systematically collected during the 2017 excavation and discuss Dian subsistence in light of the recently accumulated archaeobotanical record.

## The site of Dayingzhuang

### Environment and excavation

**Fig. 2:**
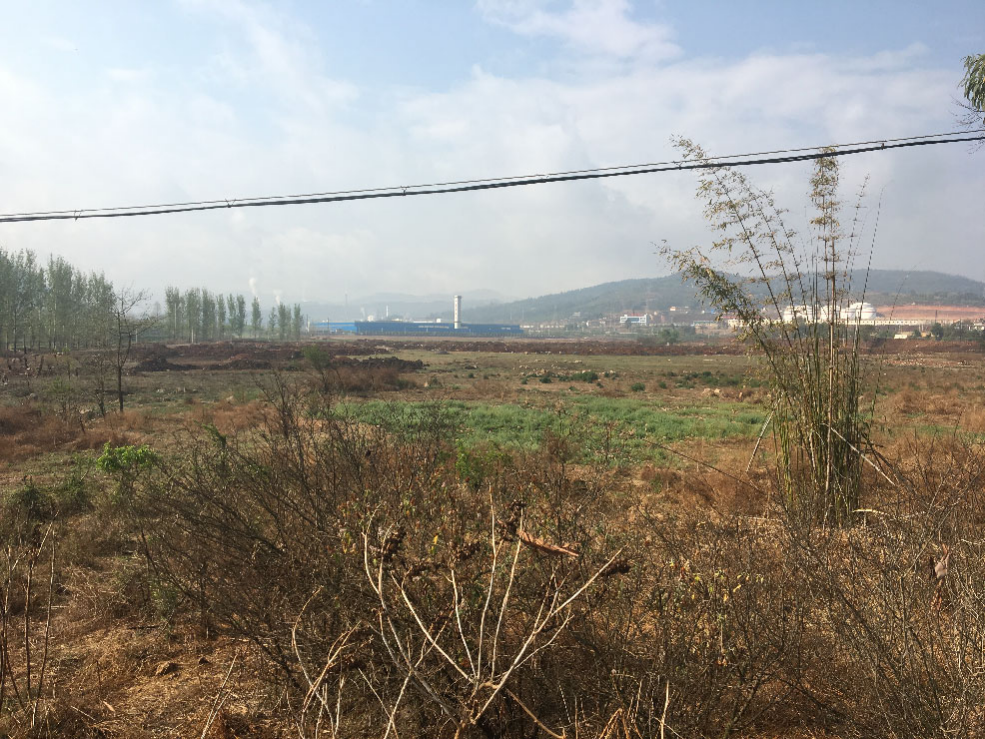
Modern day surroundings at the site of Dayingzhuang, facing North. Photo by Rita Dal Martello

The Dian Basin takes its name from the Lake Dian, the largest water reservoir in Yunnan province, and the sixth largest of the whole country, with a total area of 298 km^2^. The Dian Basin sits in the middle of the Yungui Plateau, which presents an average altitude of 1886 m asl. Modern climate conditions are very mild, and Kunming is known as the “city of the eternal spring” due to its year-round spring-like conditions. Here, annual average temperature is 15.7 °C, temperature differences between seasons is small; there are at least 240 frost-free days, about 2200 h of sun per year, and an annual average precipitation of 960 mm occurring mostly between the months of May and August, with a recorded average precipitation of 203.1 mm in July only in the last 30 years (Xiao et al. 2020; Zhao 1986). Dayingzhuang is located c. 37 km southwest from Kunming, and only 13 km from the north-western bank of the Lake Dian, at an altitude of 1920 m asl (N 24°84′; E 102°53′, Fig. 1; Fig. 2). This creates modern weather conditions slightly colder than at Kunming, with an annual average temperature of 13.2 °C (coldest attested temperatures are around − 8 °C in January and hottest attested temperatures around 28 °C in July). A rather high average daily thermal excursion of around 9.4 °C is reported; frost-free days varies between 180 and 220 per year (Zhao 1986). Modern agricultural production in the Yungui plateau is rich, with double cropping of rice and winter wheat (Zhao 1994; Bray 1984; NBS 2019).

Paleo-environmental studies from lake sediments and pollen remains in Yunnan indicate that during the mid-2nd millennium BC the summer monsoon intensity decreased, temperatures cooled, and the broad-leaved forest declined. Lake sediments from the province show an increased presence of disturbance taxa, possible indication of greater human activity and modification of the natural landscape (i.e. Xiao et al. 2020; Dearing et al. 2008; Shen et al. 2005). A sharp drop event in the monsoon intensity took place at around 1500 BC (Dykoski et al. 2005; Hillman et al. 2017); this brought the environmental conditions of the province close to those of the present day, as described above. Recent pollen analysis from Lake Dian sediments has shown an increased fire activity as well as an increased presence of Poaceae remains over the 1st millennium BC (Xiao et al. 2020).

**Fig. 3:**
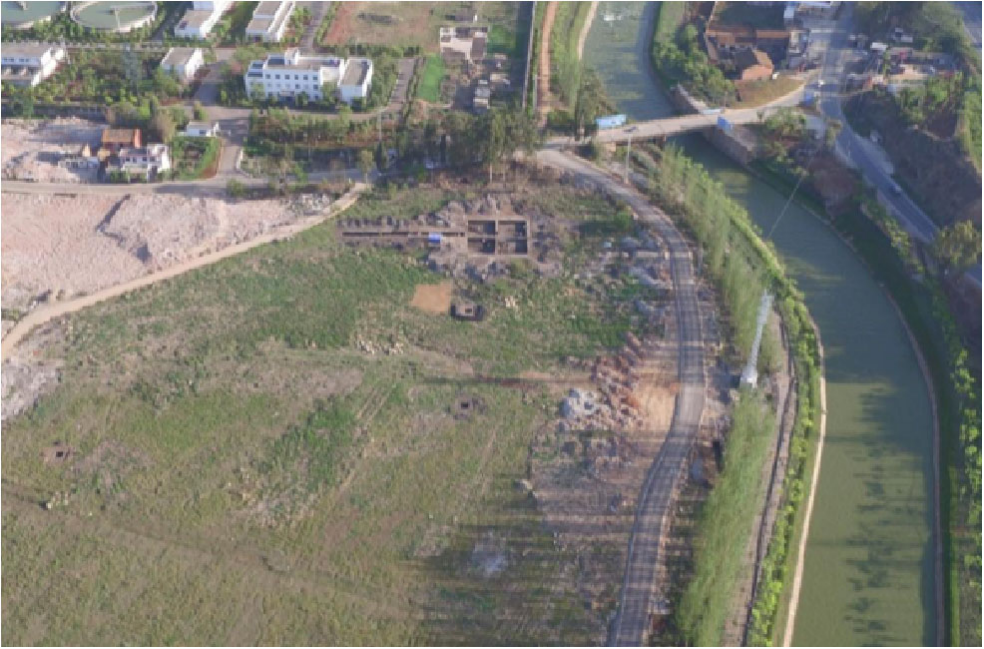
Arieal view of the excavation at Dayingzhuang. Photo by Li Xiaorui

**Fig. 4:**
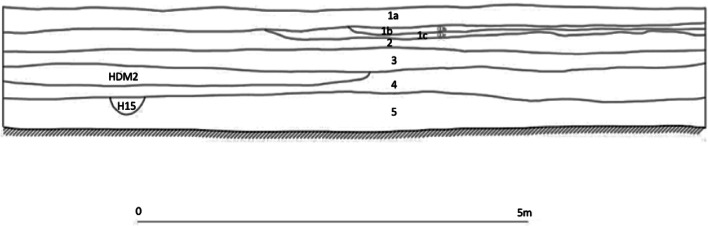
Trench TN1E2 north wall profile section showing stratigraphy and cultural layers individuated at Dayingzhuang. Provided by Li Xiaorui

Dayingzhuang is a shell-midden site first discovered in 2010 during a 3-year archaeological survey led by the Yunnan Province Institute of Archaeology, Michigan University, and the Anthropology Museum in Toronto (YPICRA et al. 2015; Yao and Jiang 2012). The imminent construction of a tobacco factory at Dayingzhuang led to a rescue campaign being carried out between March and May 2017. A total area of 500 m^2^ was excavated divided in 4 square trenches of 10 × 10 m^2^ each, and five additional trenches measuring respectively one 2 × 30 m^2^, one 2 × 14 m^2^, and three 2 × 2 m^2^ (Fig. 3). Five total stratigraphic layers including two modern layers were individuated and reached a total depth of c. 2.8 m (Fig. 4). The total estimated size of the site is between 40,000 and 100,000m^2^ (YPICRA et al. 2015).

### Chronometric results: dates and sequences

**Fig. 5:**
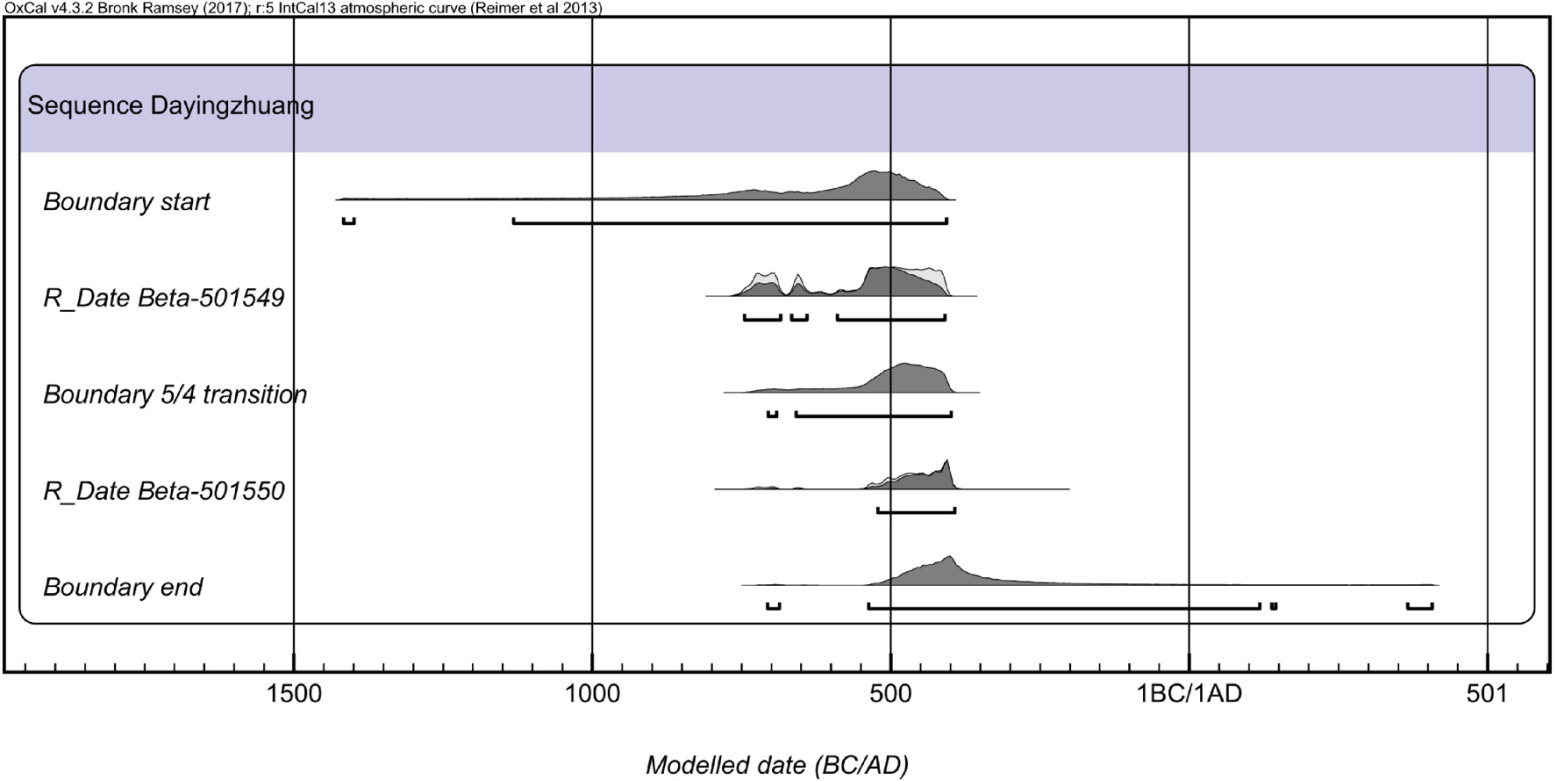
Bayesian model of the calibrated radiocarbon dates from the 2017 excavation at Dayingzhuang. Made with Oxcal v.4.3.2 (Bronk Ramsey, 2017)

During the 2010 Dian Basin survey, one radiocarbon date for the site of Dayingzhuang was published in YPICRA et al. (2015), furnishing a date of 780–550 BC (YPICRA et al. 2015: 158; see Table 1). However, the dating material used for this date is unknown, presumably bulk charcoal. Following the 2017 excavation and the collection of archaeobotanical samples, further material was available for dating, and in summer 2018 wheat grains from contexts 2017YHD(2)S4; 2017YHD(4)S4; 2017YHD(5)S1, corresponding to layer 2, 4 and 5, were submitted to the Beta Analytic Ltd. London BioScience Innovation Centre for AMS radiocarbon dating. While a wheat grain from Layer 2 appeared intrusive (ca. 100 BP), the other two dates fell in the calibration plateau, between ca. 750 BC and 390 BC, like the charcoal dated from the survey (Table 1; Fig. 5). Due to the strong stylistic differences in ceramics (see below) between layer 5 and 4, the contexts from layer 5 have been considered as part of a first period of occupation; at the moment, no direct dates are available for layer 3, and contexts from layer 4 and 3 have been considered together as part of period 2/3 in the analysis below. Based on the limited evidence from calibrated dates, we suggest that the earlier period falls sometime between 750 and 450 BC, while the Layer 4 of the later phase falls between ca. 500 BC and 390 BC (Fig. 5).

**Table 1:** Radiocarbon dates from Dayingzhuang, indicating context of provenance, material dated, and lab code (YPICRA et al. 2015; Dal Martello 2020). From Dal Martello 2020

Context	Material	Lab code	Cal. Date BP	95.40% (Bayesian model agreement index)
2010 survey	Unidentified charcoal	Beta-312946	n/a	780–550 cal. BC
2017 excavation				
Layer 2 (modern)	Wheat grain	Beta-501549	100 ± 30	18th or nineteenth century AD
Layer 4	Wheat grain	Beta-501550	2380 ± 30	522–393 cal. BC (A = 108.1%)
Layer 5	Wheat grain	Beta-501549	2430 ± 30	746–410 cal. BC (A = 102.2%)

### Site description and material culture

#### Features

Thirty-five ash pits, 4 dwellings, 2 activity floors, 5 *jicao* “wall foundations” (most likely dwellings of which limits could not be distinguished), and 1 hearth were excavated during the 2017 campaign. Earlier houses cut into the bedrock; these were mostly oval in shape with deep foundations and local archaeologists hypothesised each had a closed pavilion structure. Later houses differ by having a mostly rectangular perimeter with a clear set of postholes along each side, characteristic of wattle and daub structures. Postholes were also found inside the so-called *jicao* features. Finally, 5 *hedao* (Chinese for river) fluvial contexts were also found; these are deposits within the vestiges of an ancient riverbed, which has been identified as the pre-Han riverbed of the modern Tanglang, now flowing just outside the excavation area (see Fig. 3).

#### Ceramics

**Fig. 6:**
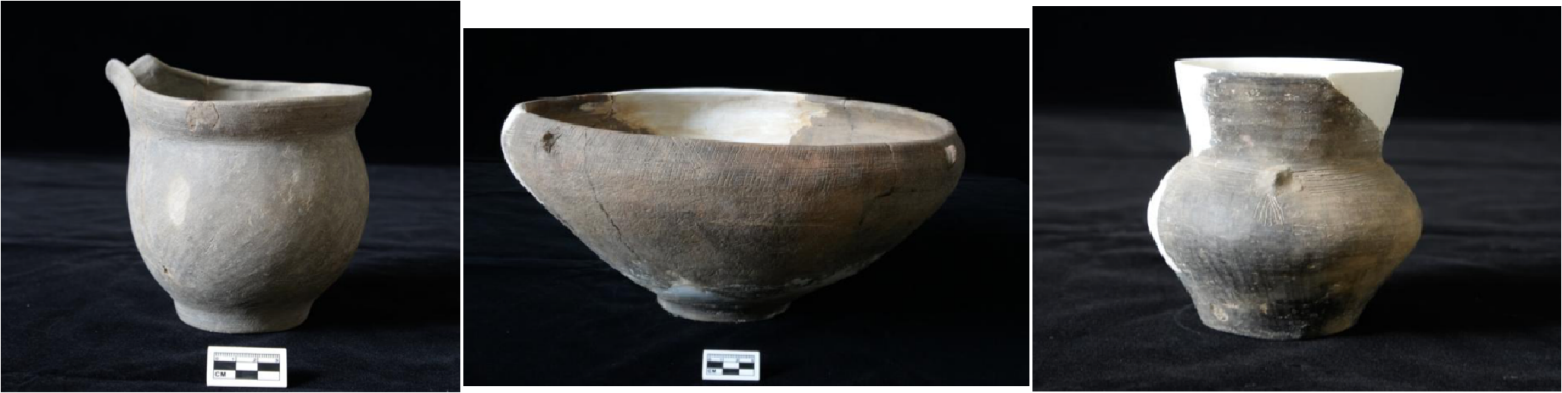
Ceramic vessels unearthed at the site of Dayingzhuang. Left to right: *guan* jar with spout; basin; high neck *guan* jar. Photos by Li Xiaorui

Pottery vessels found at Dayingzhuang include *fu* cauldrons, *bo* bowls, *guan* jars, high neck *guan* jars, and *pen* plates (Fig. 6). These are characterised by coarse, reddish or greyish temper, with numerous rice husk inclusions trapped in the temper. The decorations are characterised by incised wave (*shuibowen* 水波纹), comb (*zhiwen* 栉纹), bow-string (*xianwen* 线纹), and corded patterns (*shengwen* 绳纹), which are characteristic of Dian ceramics. According to stratigraphic and ceramic typology, there have been distinguished at least three cultural phases:

- Early phase, corresponding to layer 5; ceramic remains are mostly of coarse grey temper and show a high presence of incised decoration. Vessel assemblage is dominated by *guan* jars, some with pouring sprouts. Oval, pavilion-type dwellings.
- Middle phase, corresponding to layer 4; appearance of coarse, red temper pottery and decline of grey temper, vessel assemblage dominated by *bo* bowls. Appearance of wattle and daub type dwellings.
- Late phase, corresponding to layer 3; vessel assemblage dominated by incensed circles (*tongxinyuan* 同心圆纹*)* decorated *pen* plates, *bo* bowls decrease.

#### Stone and other implements

Stone tools found at Dayingzhuang include polished *fu* axes, *ben* adzes, and *bing-*round stone fragments*,* grindstones, etc. Bone and shell implements were also found, including needles, pins, and scrapers. Finally, some processed agate stone fragments and cowrie shells have been unearthed. The cowrie shells showed holes in their upper extremity, indicating they might have had an ornamental function.

#### Metal objects

**Fig. 7:**
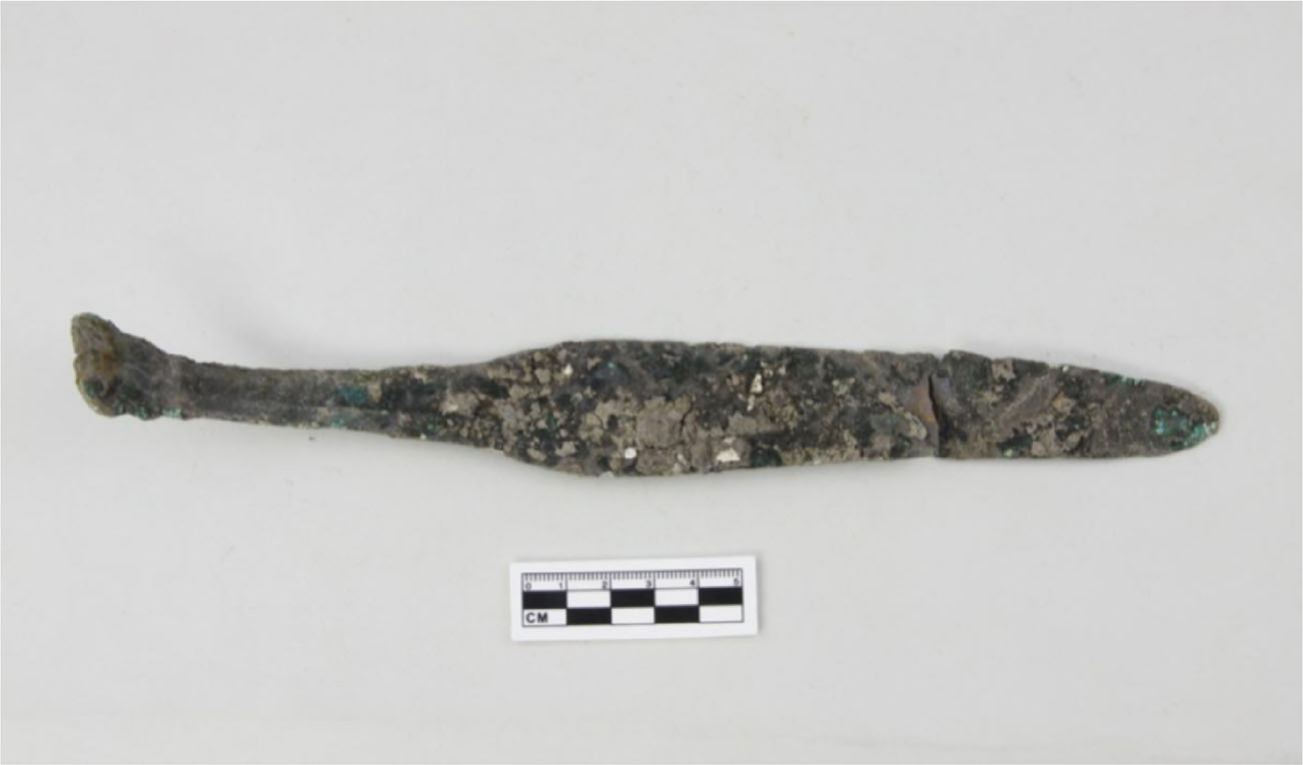
Metal dagger unearthed at the site of Dayingzhuang. Photo by Li Xiaorui

A dagger (Fig. 7), a plain, wide and flat bracelet, and some metal chips were recovered during the 2017 excavation; metallurgical slags were also reported during the 2010 survey (YPICRA et al. 2015). The limited occurrence of bronze objects at the site of Dayingzhuang is not surprising as Dian bronzes are in fact mostly associated with (wealthy) burials, such as those at Shizhaishan, and generally found in higher quantities in cemeteries, not so frequently found at settlement sites.

#### Faunal remains

Numerous bone tools have been found, including remains of pig, cattle, horse, deer, and fish, but no systematic zooarchaeological analysis has been undertaken on these remains yet. The gastropod species *Margarya melanioides,* a large edible aquatic snail, constitutes the majority of the shell remains found in the mound layers (YPICRA et al. 2015).

## Materials and methods

### Macro-botanical remains

Over 130 archaeobotanical samples were collected for flotation from each feature across the whole excavation area of the site; from cultural layers in trench TN2E2 (the southwestern 10x10m^2^ trench,^1^ and additional cultural layers samples were collected at the cross of the four 10x10m^2^ trenches, and labelled GJZ (*guanjianzhu* 关键柱 meaning “control column”), followed by the layer number. Each sample had a bulk soil volume of 20 L. All samples were manually floated at the site during excavation with the use of a bucket, and the float was collected with a 0.3-mm mesh into a cotton cloth bag, finally dried naturally in the shade.

All samples collected were scanned at the Yunnan Province Institute of Cultural Relics and Archaeology- Archaeobotany Laboratory under a Leica low power binocular microscope at magnification up to X40. Of the scanned samples, 32 appeared particularly rich in archaeobotanical remains and were therefore selected to be fully analysed at the UCL-Institute of Archaeology-Archaeobotany Laboratory. The selected samples covered a complete sequence top to bottom, including samples from layer 2 to layer 5, and associated features. However, AMS radiocarbon dating on wheat grains from layer 2 furnished a modern date (see above), therefore contexts corresponding to this layer have been excluded, and a total of 30 samples, corresponding to contexts from layers 3 to 5, are presented here.

### Phytolith remains

Phytoliths samples were collected vertically every 10 cm from the GZJ column without performing sieving or any other kind of soil processing; each sample was put into a plastic bag and let dry in the shade. About 10 g of soil per each sample was brought back to UCL. 12^2^ individual samples were selected to be processed and analysed). Even numbers between samples #16 and #34 (excluding #28) were selected. Samples #3 and #10 were also selected to provide a modern vegetation baseline (Table 3 below). Laboratory extraction was carried out at the UCL Institute of Archaeology-Phytoliths Laboratory following an adaptation of the protocols of A. M. Rosen (1999; see S1).

Each phytolith slide was examined with the use of a biological binocular microscope with magnifications up to 400×. Phytoliths were counted to reach a minimum of 300 single cells and 150 multi-cells counts respectively per each slide. Phytoliths were classified according to the morphotype divisions outlined in Piperno (2006) and Madella et al. (2005).

## Results

### Macro-botanical remains

#### General features of the assemblage and key economic taxa

Only charred archaeobotanical remains were recovered from the Dayingzhuang samples, but not in great quantity, as evident by the low density of items/l, with negligible difference between the two periods (Table 2). This is most probably due to modern agriculture and erosion activity from the nearby Tanglang River. A total number of 1070 identified remains, belonging to 14 families, and over 20 individual species, have been recovered from the samples analysed (Table 2).

**Table 2:** Summary of flotation samples and plant macro-botanical remains by period and context type

	Period 1- Layer 5750–405 cal. BC	Period 2/3- Layer 4/3727–393 cal. BC
Volume floated (litres)	360	380
No. of samples	16	14
Cultural layers	4	2
Ash pits	7	8
Houses	3	4
*Hedao*	2	0
No. of identified species	19	16
Cereals		
*Triticum aestivum*- wheat grains	243	65
*Triticum aestivum*- grain fragments	98	8
*Triticum aestivum*- rachises	–	1
*Triticum aestivum*- immature grains	–	1
*Oryza sativa*- rice grains and embryos	32	21
*Oryza sativa*- rice grain fragments	41	15
*Oryza sativa*- spikelet bases	23	–
*Oryza sativa-* immature grains	7	–
*Setaria italica*- foxtail millet grains	16	17
*Setaria italica*- foxtail millet immature grains	3	1
*Panicum miliaceum*- broomcorn millet grains	18	8
Indet millet fragm.	2	–
*Hordeum vulgare*- barley grains	1	5
*Hordeum vulgare*- rachis	–	1
*Hordeum vulgare*- immature grains	–	1
Other possible cultigens		
*Chenopodium* sp.	41	312
*Euryale ferox*- foxnut fragments	8	35
cf. *Castanea* sp.- chestnut	1	–
Indet. Acorn	3	3
*Zanthoxylum* sp.- Sichuan pepper	–	3
Seeds of field weeds		
Grasses & seeds of dryland cultivation		
*Pennisetum* sp.	2	–
*Vicia* sp.	2	1
Fabaceae- indeterminate	2	–
*Solanum* sp.	1	–
*Portulaca* sp.	–	2
Euphorbiaceae- indet.	–	1
Sedges- &seeds of wetland cultivation		
*Schoenoplectus* sp.	–	1
*Rumex* sp.	2	3
*Alisma* sp.	1	–
Other weeds- seeds of both dry and wetland cultivation		
*Echinochloa* sp.	1	1
Apiaceae- indet.	1	–
Wild species		
*Ilex* sp.	–	1
Rosaceae- indet.	3	–
Indeterminate small seeds	6	5
Total macro-remains	558	512
Density of items/l	1.55	1.28

Identifiable remains were categorised in the following groups:

- Cereals, including wheat-*Triticum aestivum*, barley*-Hordeum vulgare,* rice-*Oryza sativa*, Chinese millets, both foxtail millet-*Setaria italica* and broomcorn millet*-Panicum miliaceum*, and indeterminate millets;
- Other possible cultigens (including *Chenopodium*, nuts, and fruits and other wild species);
- Seeds of likely field weed species.

#### Cereal crops

**Fig. 8:**
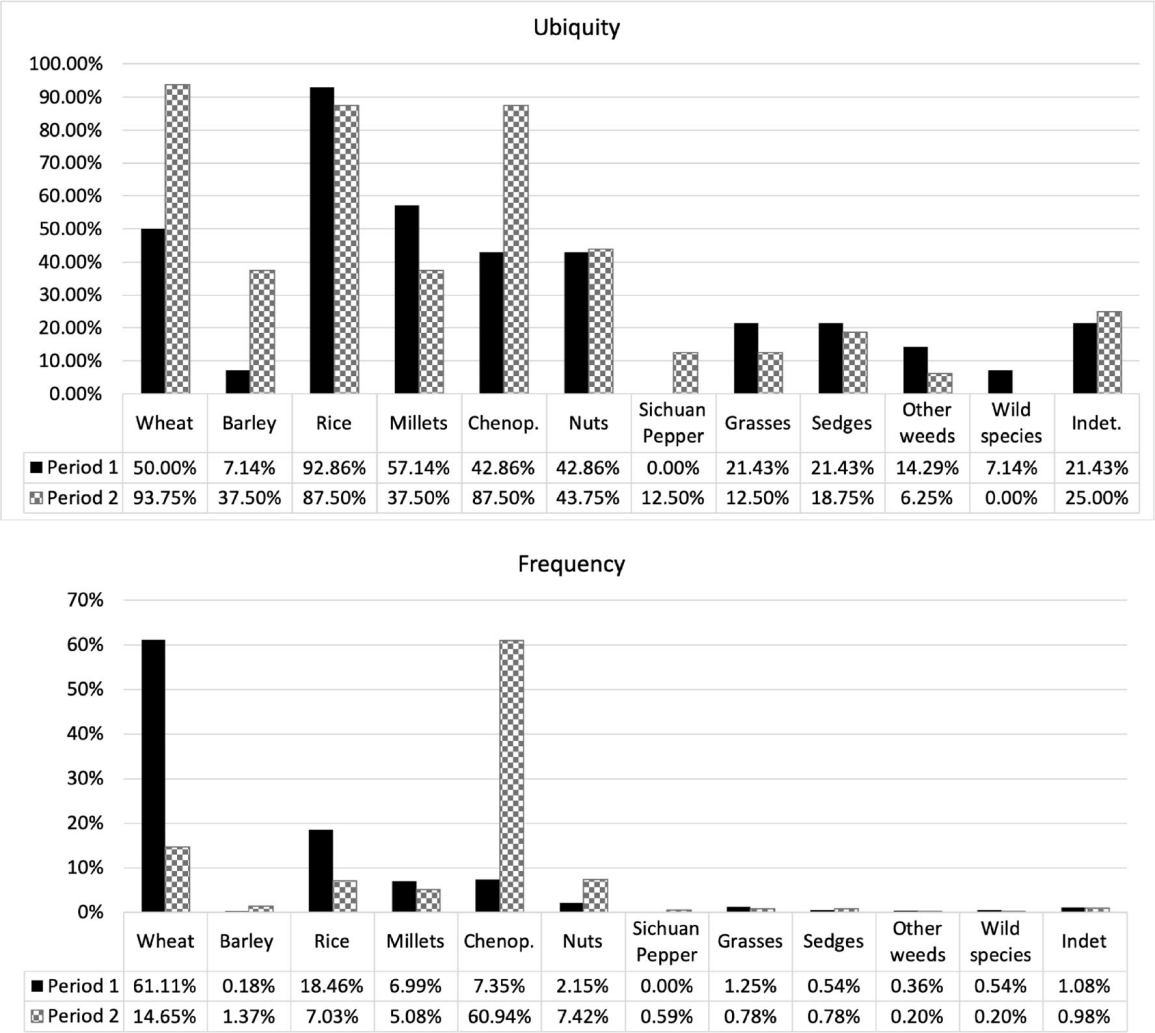
Ubiquity (top) and frequency (bottom) index of archaeobotanical remains at Dayingzhuang

Five different species of cereal crops were recovered from the Dayingzhuang samples, including Chinese domesticated rice (*Oryza sativa*) and millets (*Setaria italica* and *Panicum miliaceum*), and western domesticates, wheat (*Triticum aestivum*) and hulled barley (*Hordeum vulgare*, Fig. 16: 1–6)*.* Charring normally removes any trace of the barley hulls, but grain shape surface features allow hulled barley to be identified in this case. Naked barley grains have a rounded cross section and fine transverse wrinkling on well-preserved grains, whereas hulled barley are angular in cross-section with longitudinal ridges on the surface. The presence of asymmetric grains indicates the presence of six-row hulled barley (*Hordeum vulgare subsp. vulgare,* syn. *H. hexastichum* L.). Cereals altogether constitute c. 86% of the total identified remains for period 1; but decrease to only c. 28% for period 2/3 (Fig. 8). All other cereal species except for barley, decrease during the later period of occupation. With the exclusion of wheat, frequency and ubiquity index for cereals show a strong correlation (Fig. 8).

**Fig. 9:**
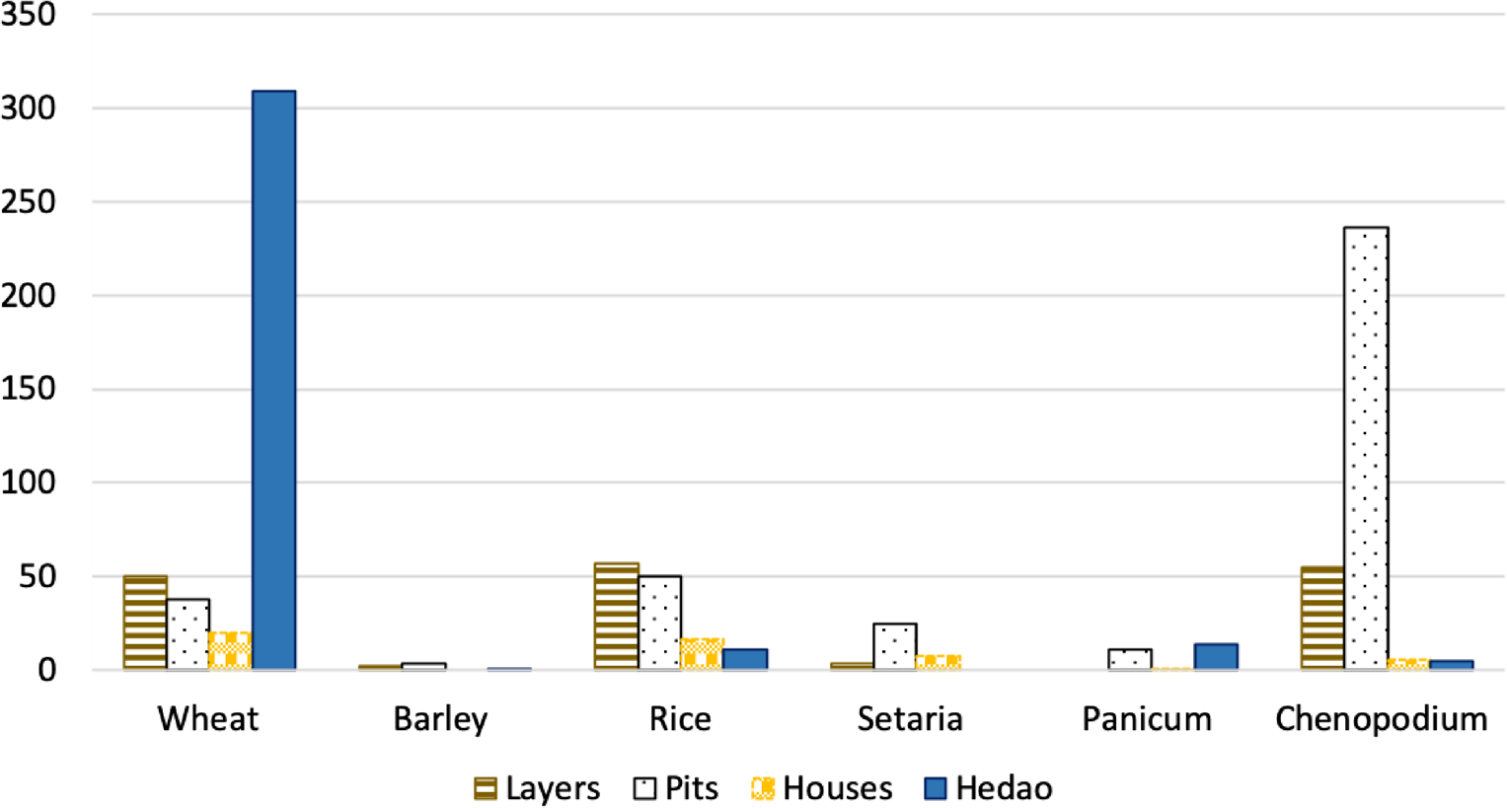
Cereal remains context analysis at Dayingzhuang, with indication of absolute counts. Hedao refers to alluvial/river adjacent contexts

The very low ubiquity of wheat remains from period 1 is due to the high concentration of wheat remains from fluvial context *Hedao* 1, where 307 out of 416 total wheat remains (including whole grains, immature grains, grain fragments and rachis) were found (Fig. 9). This type of context is not present in period 2/3 (see S2).

#### Wheat and barley

Wheat and barley have been found in c. 80% of the samples analysed, and account for c. 40% of the total archaeobotanical remains from the Dayingzhuang samples. However, barley only constitutes c. 2% of the total identified remains. Chronologically, wheat remains constitute the main species recovered during period 1, but decrease sharply in period 2/3; barley instead increases a little in period 2/3 (Fig. 8). Density of wheat remains per litre floated is 0.63/L.

**Fig. 10:**
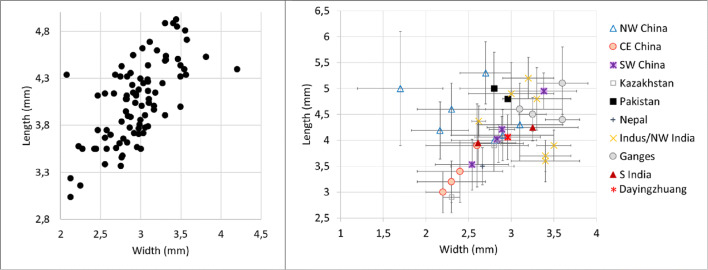
(Left) Scatterplot of L/W ratio of Dayingzhuang wheat (Triticum aestivum) grains. (Right) Averages L/W ratio from published wheat measurements dataset from countries in Asia, red star indicates Dayingzhuang (data from Dal Martello 2020; Xue 2010; Jin 2013; Li and Liu 2016; Zhou et al. 2020; Spengler et al. 2014; Spengler et al. 2013; Asouti and Fuller 2008; Fuller 2000; Saraswat 2004; Vishnu-Mittre 1982; Chanchala 2002; Pokharia et al. 2011)

**Fig. 11:**
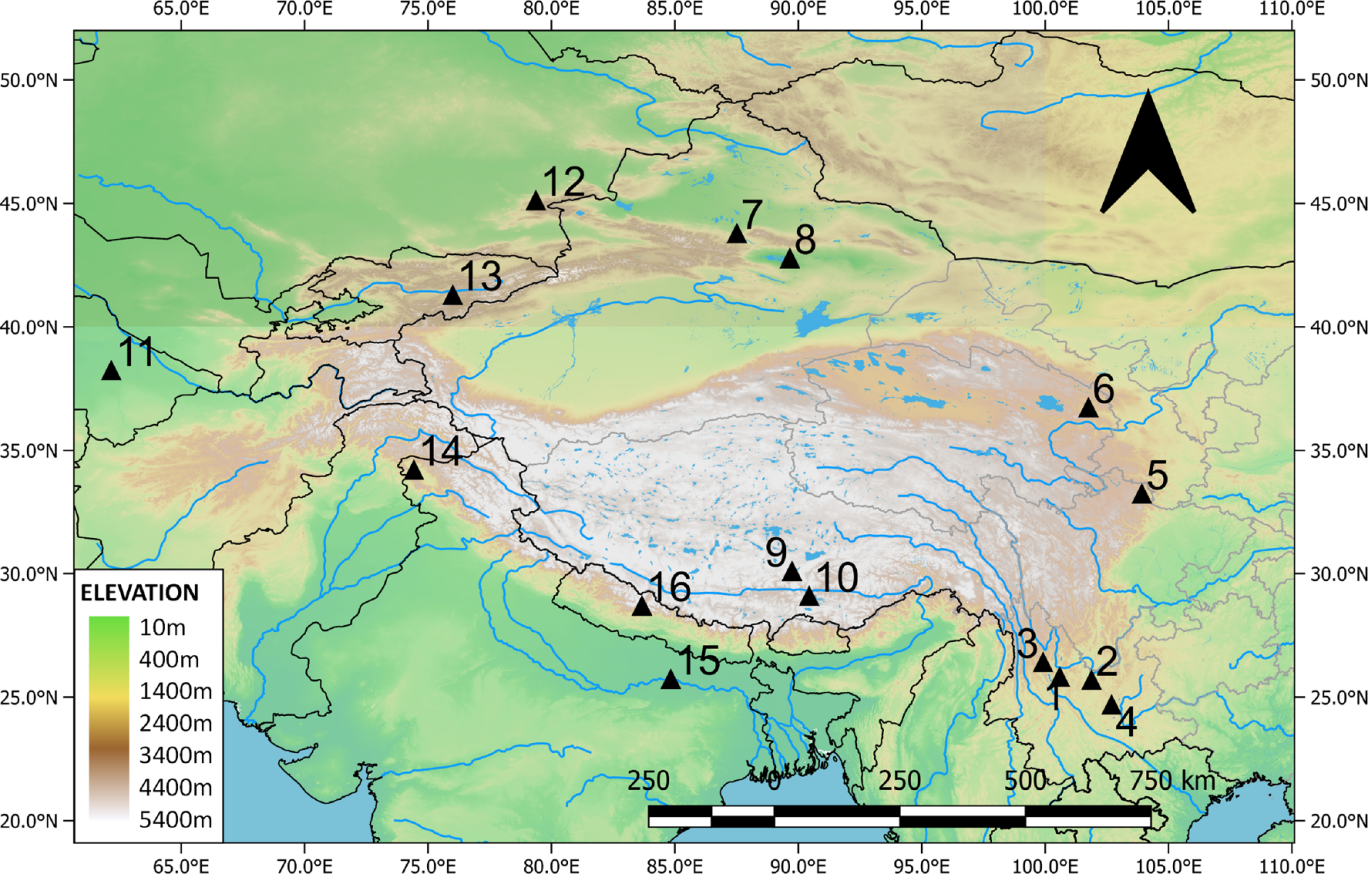
Map showing sites mentioned in the discussion, sites 3-16 report the presence of wheat and/or barley grains, measurements from these sites are plotted for comparison to those from Dayingzhuang (Figs 10, 12). 1 Baiyangcun; 2 Dadunzi; 3 Haimenkou; 4 Shangxihe; 5 A’shaonao; 6 Xiasunjiazhai; 7 Sidaogou; 8 Yanghai; 9 Khog gzung; 10 Bangtangbu; 11 Ojakly; 12 Tasbas; 13 Aigyrchal-2; 14 Kanispur; 15 Chirand; 16 Chokhopani. Made with QGIS 3.10.10

**Fig. 12:**
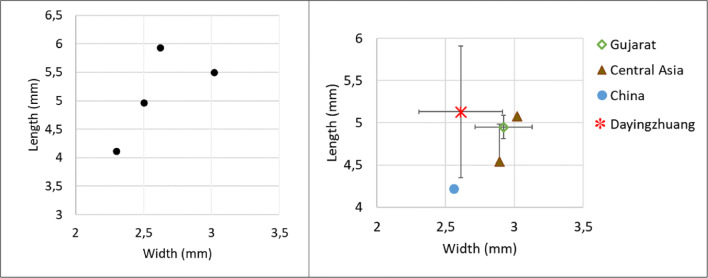
(Left) Scatterplot of L/W ratio of Dayingzhuang barley (Hordeum vulgare) grains. (Right) Averages L/W ratio from published barley measurements dataset from countries in Asia, red star indicates Dayingzhuang (data from Pokharia et al. 2011; Spengler et al. 2013, 2014; Zhou et al. 2020)

Ninety grains of wheat were measured (Table S3), and these were on average 4.06 mm long, with a standard deviation of 0.43; 2.95 mm wide (standard deviation of 0.38); and 2.53 mm thick (standard deviation of 0.30). Wheat L/W at Dayingzhuang was 1.38 mm, with a standard deviation of 0.14 mm (Fig. 10; Table S3). We compared these to a set of published measurements from across South, Central and East Asia (for a map of sites see Fig. 11). Dayingzhuang wheat falls amidst wheat from other sites in Southwest China, some assemblages from northwest China and from Central Asia (Kazakhstan), and further away from published assemblages in northern India or Central/Eastern China. Only 4 grains of barley from the Dayingzhuang samples were well preserved enough to be measured, and these were on average 5.13 mm long, 2.61 mm wide, and 2.12 mm thick; average L/W was 1.96 mm, with a standard deviation of 0.21 mm (Fig. 12; Table S3).

#### Rice

**Fig. 13:**
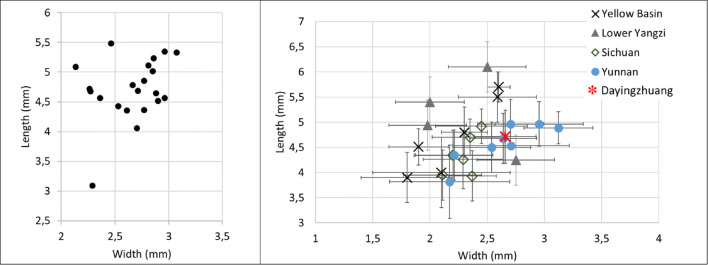
(Left)Scatterplot of L/W ratio of rice (Oryza sativa) grains.(Right) Averages L/W ratio from published rice measurements dataset in China, red star indicates Dayingzhuang (data from Crawford et al. 2006; Tang 1999; Li 1994; Lee and Bestel 2007; Tang et al. 2003; Huang and Zhuang 2000; Zheng et al. 2004; Fuller et al.^ 2014^; Shanghai Museum 2014; Fuller et al. 2010; Zhao 2003; D’Alpoim et al. 2009; D’Alpoim et al. 2013; Zhao 2011; Yang 2016; Wang 2014; Pei 1998; Zhang and Wang 1999; Liu et al. 2007; Li and Liu 2016; Deng 2016 unpublished). Remade from Dal Martello 2020

Rice remains have been found in 90% of the samples analysed at Dayingzhuang, however they only constitute c. 13% of the total identified remains. Rice remains constitute c. 18% of the total identified remains for period 1, but decrease to only 7% for period 2/3, even though the ubiquity of rice remains for both periods is quite high. Rice density is 0.17/L. A total of 21 rice grains were measured, and these were on average 4.71 mm long, with standard deviation of 0.52 mm; 2.65 mm wide, with standard deviation of 0.27 mm; and 2.31 mm thick, with standard deviation of 0.33 mm (Table S3). Average L/W was 1.78 mm, with a standard deviation of 0.24 mm (Fig. 13). According to measurement guidelines set out in Harvey and Fuller (2005) and Castillo et al. (2016), rice L/W of > 2.2 can be classified as *Oryza sativa *subsp.* indica*, and L/W of < 2 is generally considered *Oryza sativa *subsp.* japonica*. Among the rice grains measured from Dayingzhuang, only 2 grains measured > 2.2 mm in L/W, therefore, rice at Dayingzhuang has been classified as *Oryza sativa *subsp.* japonica*. Moreover, no morphologically wild rice grains or spikelet bases have been recovered from the Dayingzhuang samples.

#### Millets

Foxtail and broomcorn millets were both found in the samples analysed; the two millets account for only c. 6% of the total identified remains at Dayingzhuang; although foxtail millet is present in slightly higher quantity than broomcorn, the differences between the two are negligible (see S2). Millet density per litre is 0.8 items/L.

**Fig. 14:**
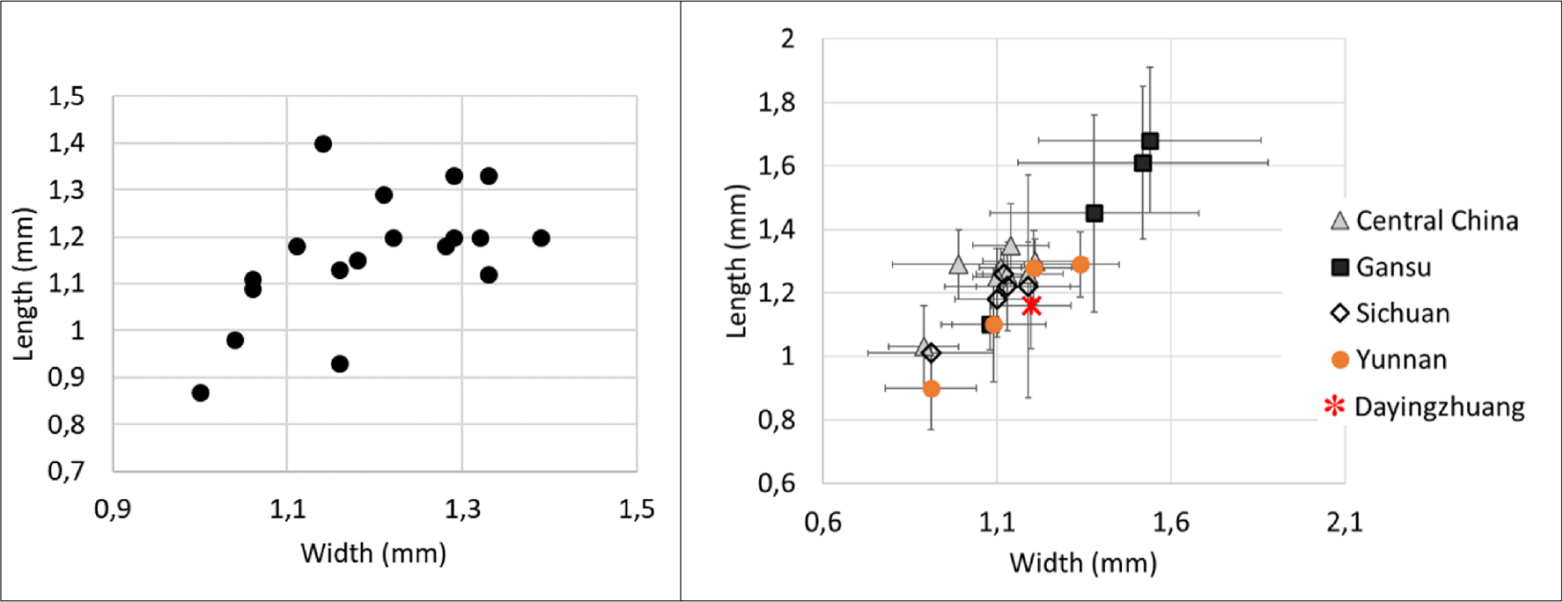
(Left) Scatterplot of L/W ratio of Dayingzhuang foxtail millet (Setaria italica) grains. (Right) Averages L/W ratio from published Setaria measurements dataset in China, red star indicates Dayingzhuang (data from Barton 2009; Yang 2016; Stevens unpublished data). Remade from Dal Martello 2020

**Fig. 15:**
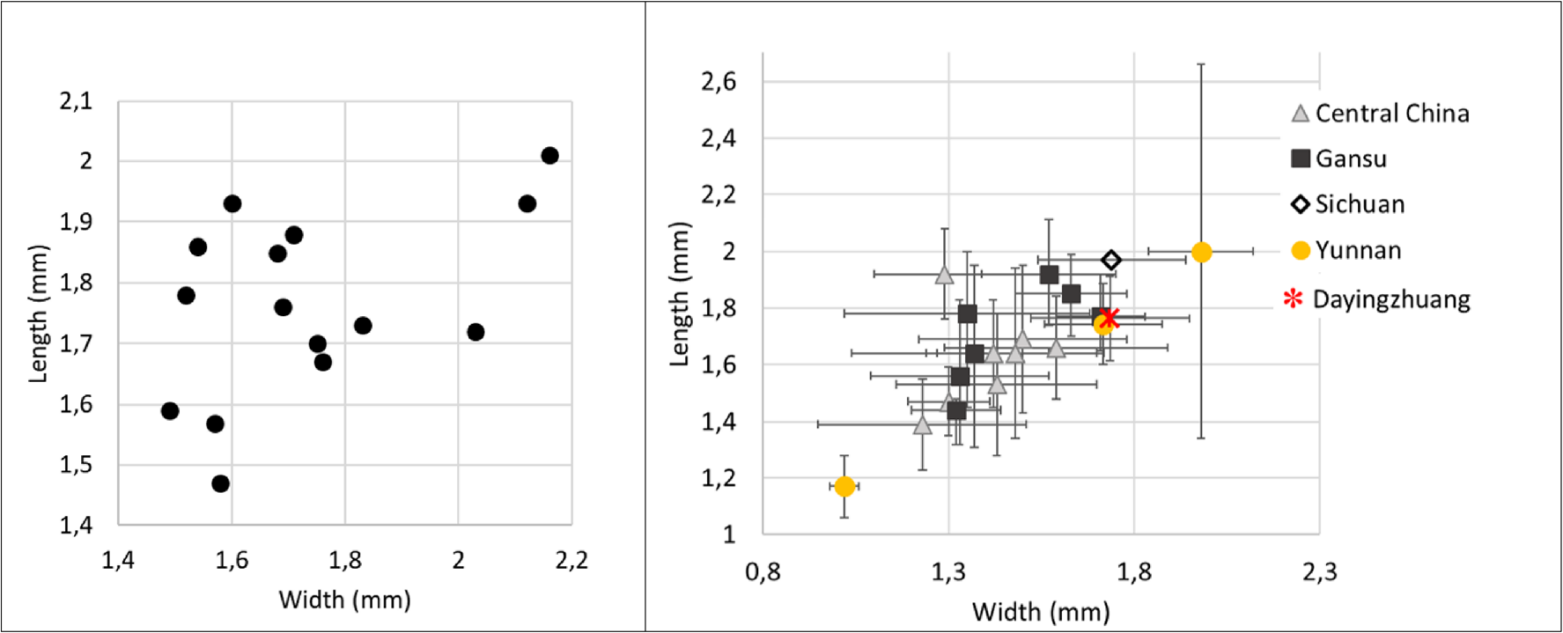
(Left) Scatterplot of L/W ratio of Dayingzhuang Broomcorn millet (Panicum miliaceum) grains. (Right) Averages L/W ratio from published Panicum measurements dataset in China, red star indicates Dayingzhuang (data from Barton 2009; Yang 2016; Stevens et al. 2020). Remade from Dal Martello 2020

Eight complete well-preserved grain of foxtail millet were measured, and these were on average 1.16 mm long (standard deviation of 0.13 mm), 1.19 mm wide (standard deviation of 0.11 mm), and 0.9 mm thick (standard deviation of 0.1 mm). The average L/W for foxtail millet was 0.97 mm (standard deviation of 0.10 mm); these are comparable to other Neolithic and Bronze Age grains from southwest China (Fig. 14). Fifteen grains of broomcorn millets were measured, furnishing an average length of 1.76 mm (standard deviation 0.15 mm). width 1.73 mm (standard deviation 0.21 mm), and thickness 1.3 mm (standard deviation 0.21 mm). Average L/W was 1.33 mm (standard deviation 0.21 mm). These also compare well with domesticated broomcorn millet identified from elsewhere in China (Fig. 15; see also Table S3; Stevens et al. 2020).

#### Chenopodium

**Fig. 16:**
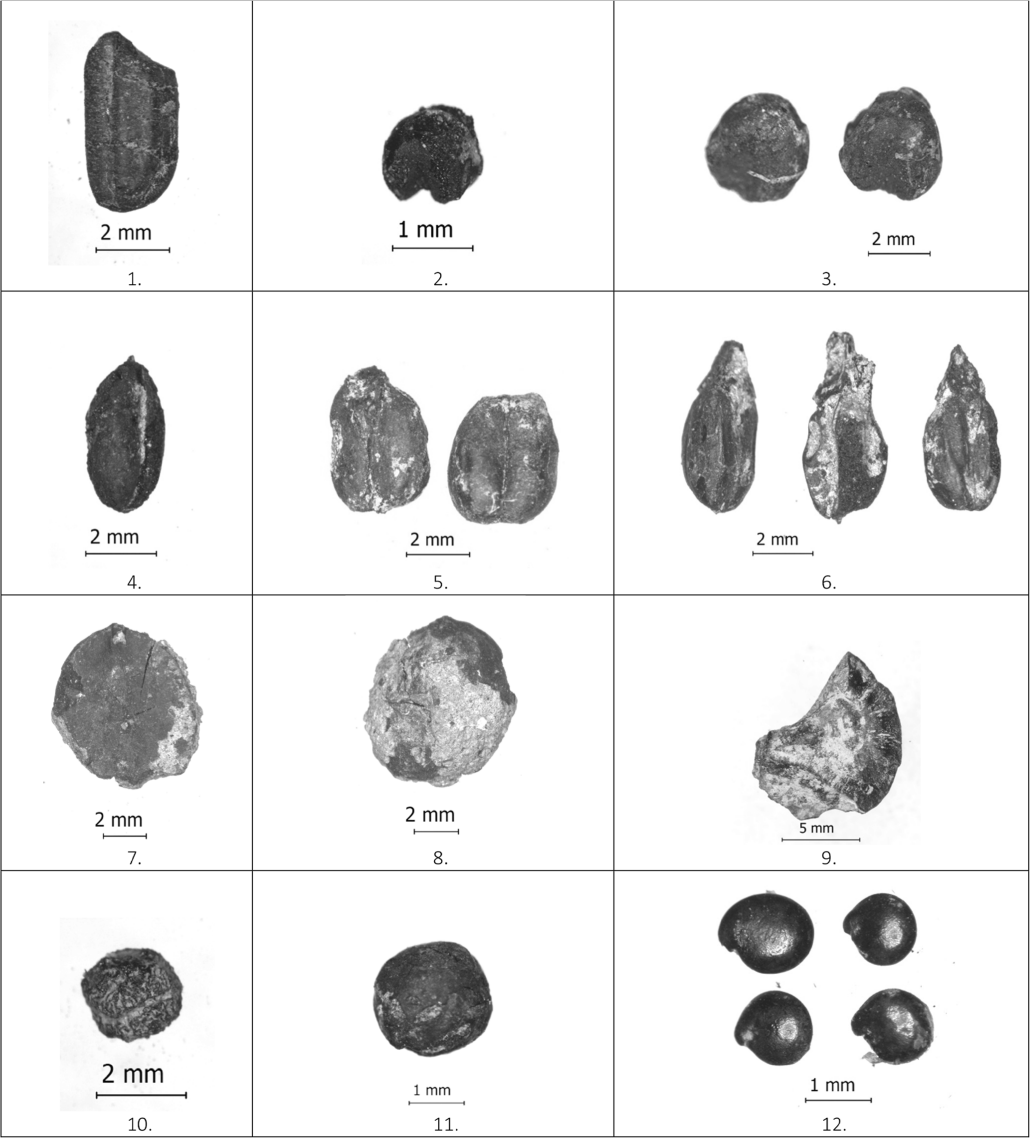
Photos of macro-botanical remains from Dayingzhuang. 1. *Oryza sativa*; 2. *Setaria italica*; 3. *Panicum miliaceum*; 4. *Hordeum vulgare*; 5. *Triticum aestivum*; 6. *Triticum aestivum*; 7-8., *Castanea* cf.; 9. Indet. acorn ; 10. *Zanthoxylum* sp.; 11. *Vicia* sp.; 12. *Chenopodium* sp. From Dal Martello 2020

At Dayingzhuang, *Chenopodium* grains have been recovered in very high quantity and ubiquity, especially from the 2/3 period of occupation; it is the second most abundant species recovered (Fig. 8; Fig. 16: 12), and the total density of *Chenopodium* grains per litre is 0.46/L. In the first period of occupation *Chenopodium* is found mostly in layer contexts; during period 2/3 instead, the species has been recovered from pit contexts, especially H8 and H11 (see Fig. 9). This trend is similar to that seen for dryland weeds at the site; however, these were recovered in extremely low quantity. Measurements on 28 *Chenopodium* grains showed average measurements comparable to modern wild *Chenopodium* (Table S3; Dal Martello 2020). It has been proposed that this species might have been cultivated at the earlier sites of Guiyuanqiao in Sichuan (dated to between c. 3100 and 2000 BC, D’d'Alpoim Guedes and Wan 2015), at Haimenkou, in Northwest Yunnan (dated to between 1600 and 400 BC; Xue 2010; Dal Martello 2020), as well as from later sites during the Han Dynasty (Yang and Liu 2009). However, very little archaeological investigation of this species’ domestication trajectory has been undertaken so far, with most publications categorising this species as a weed; therefore, until more systematic analysis is done on more archaeological assemblages, the specific role, if any, *Chenopodium* had at Dayingzhuang remains unclear.

#### Other possible cultigens

Although recovered in very low quantities, other economic species were found at Dayingzhuang (Fig. 16: 7–11), including remains of foxnut (*Euryale ferox)*, chestnut (*Castanea* sp.), unidentified remains of acorns, Sichuan pepper (*Zanthoxylum* sp.), unidentified Rosaceae, and *Ilex* sp. Among these, nut remains are found in about 40% of the samples analysed. Noteworthy is the find of *Zanthoxylum* seed, as Southwest China has been indicated as the possible domestication centre for Sichuan pepper. Recently, thanks to the more systematic use of flotation to recover macro-botanical remains during excavations in China, archaeological *Zanthoxylum* remains have been increasingly reported from sites in Southwest China (Sheng et al. 2020; D'Alpoim Guedes 2013; Li et al. 2016; Yang 2016; Wang 2014).

#### Seeds of field weed species

Not many remains of field weed species were recovered from the Dayingzhuang samples, including seeds of dryland field weeds, such as *Pennisetum* sp., and *Vicia* sp.; nutlets of wetland field weeds, such as *Schoenoplectus* sp., and *Rumex* sp., and a few grains of *Echinochloa* sp. They altogether account for only 2.15% of the archaeobotanical assemblage for period 1, and 1.76% for period 2/3. The differences between dryland and wetland weed frequency and ubiquity are negligible across different time periods, and their overall very low presence make them not statistically significant for investigating crop ecology at the site. Nevertheless, those taxa present suggest some combination of wet agriculture fields, presumably for rice, and dry cultivation, which might include millets or wheat and barley.

### Phytoliths remains: general features

A total of 11 phytolith samples, covering a complete sequence top to bottom, including 2 samples from modern layers, were analysed (Table 3). The average percentage by weight of phytoliths for the samples analysed was 6.48%; minimum percentage weight was 0.37%, (sample 30) and maximum percentage weight was 22.7% (sample 34); however, most of the samples were less than 1%, and only four had percentage weights over 9% (see Table 3).

**Table 3:** Summary of Dayingzhuang phytolith samples, with indication of their original sample number, laboratory sample ID and stratigraphic relation to cultural layers, and percentage weight of phytoliths per each sample. From Dal Martello 2020

Original sample #	Lab. Phytolith ID	Corresponding stratigraphy	% weight
3	12	Modern topsoil	0.53
10	11	Layer 2 (modern)	5.86
*12*	*10*	*Layer 3 (discarded)*	*n/a*
16	9	Layer 3	13.07
18	8	Layer 4	1.24
20	7	Layer 4	0.62
22	6	Layer 4	1.76
24	5	Layer 4	14.8
26	4	Layer 5	9.9
30	3	Layer 5	0.37
32	2	Layer 5	0.38
34	1	Layer 5	22.7

**Fig. 17:**
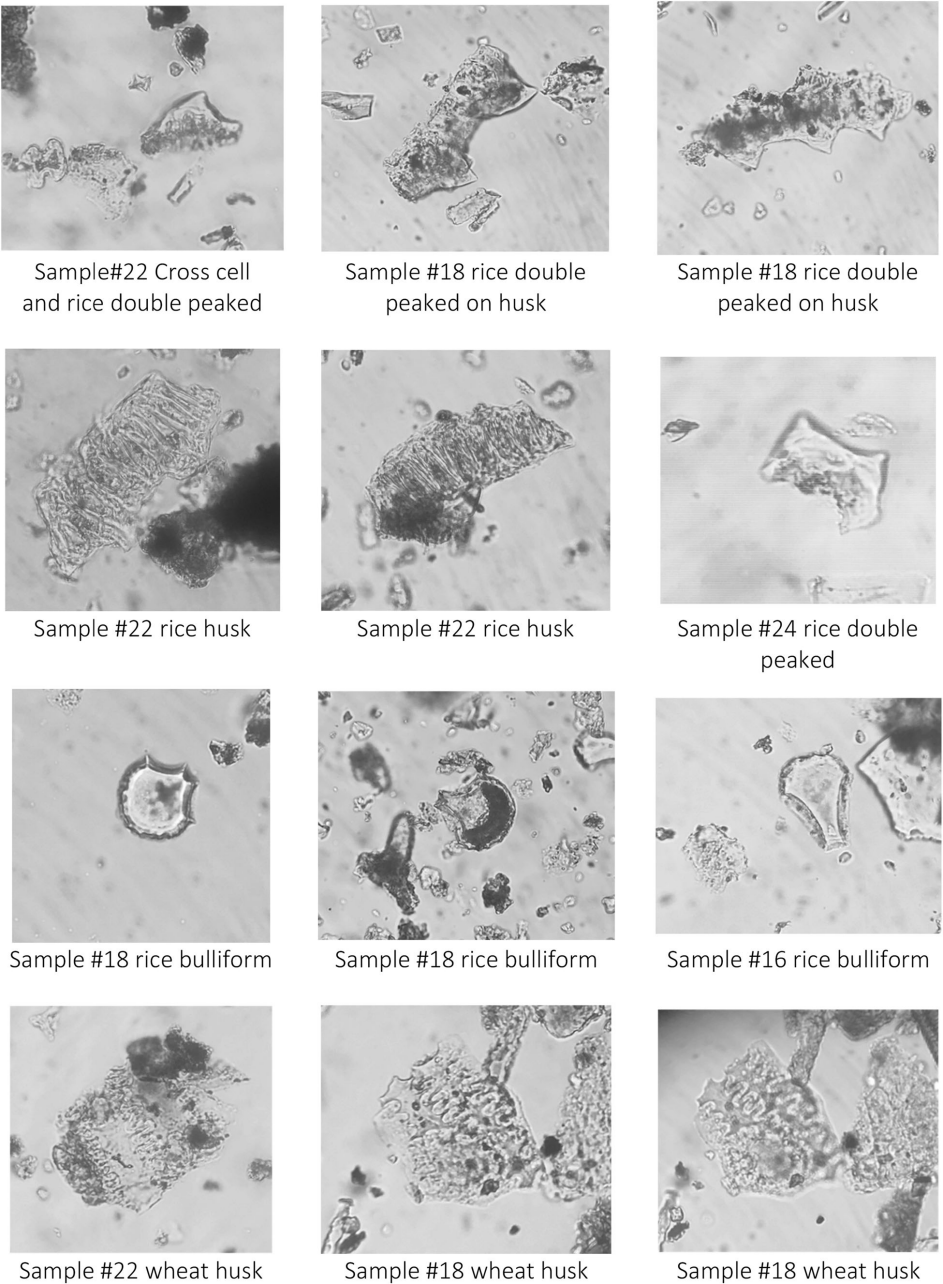
Photos of Dayingzhuang phytoliths remains. From Dal Martello 2020

**Fig. 18:**
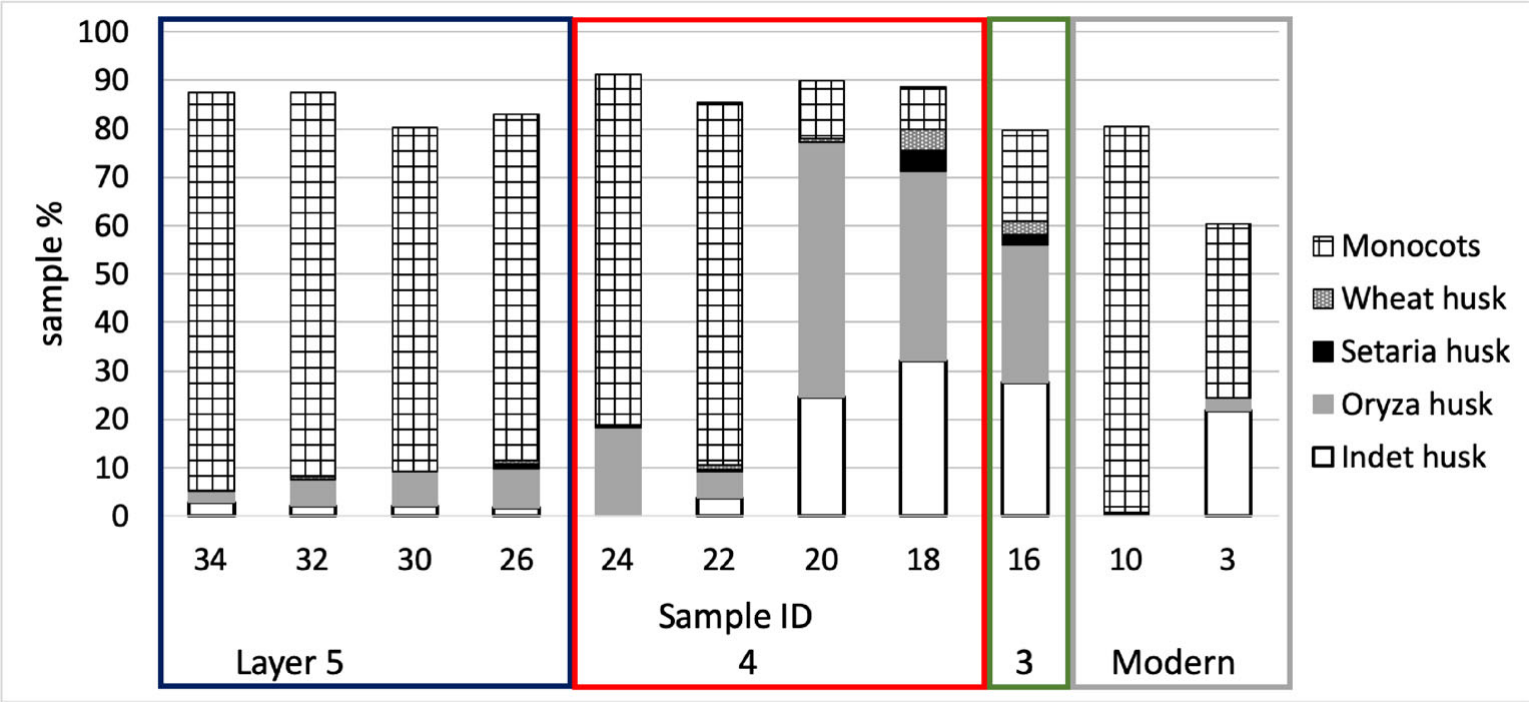
Husks remains from phytolith samples at Dayingzhuang

Monocot single cells were the most abundant phytoliths recovered from the samples analysed, accounting for 70% of the total single cells. Among the single cells, long cells are the most common type found (smooth, echinate, or dendritic), secondarily rondels and saddles. Among the multi-cell morphotypes, unidentified husk remains and *Oryza* type husks (especially *Oryza* double-peak glume cells) are the most abundant type recovered (S4; Fig. 17). *Oryza* husk phytoliths have been recovered from all samples analysed, and they have been found in especially high quantity in samples #20, #18, and #16, corresponding to layer 4 (period 2/3; Fig. 18). In these samples monocot single cells decrease to c. 8–18%. Further husk phytoliths of the cf. *Setaria* and cf. *Triticum* types have been found in 7 samples (Fig. 18).

**Fig. 19:**
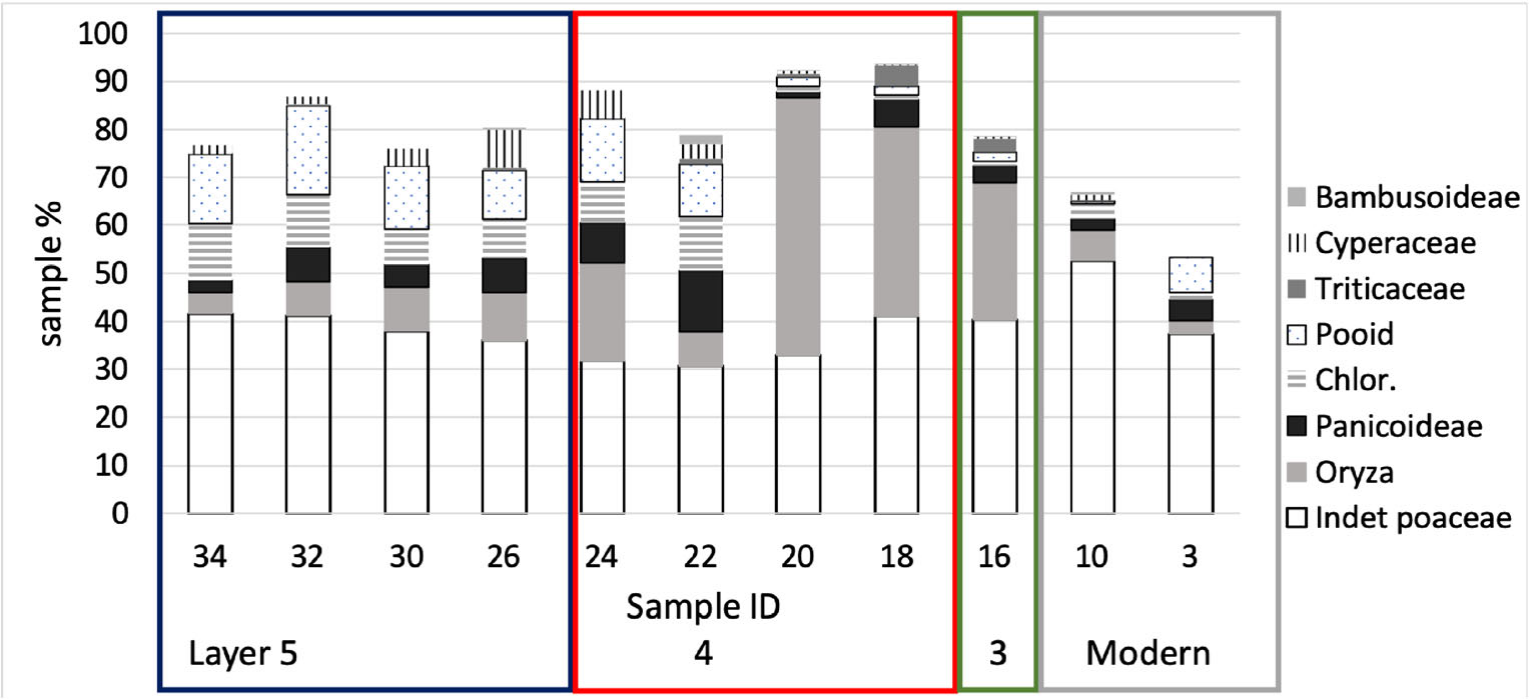
Relative frequencies of subfamilies phytoliths remains at Dayingzhuang

Other grass taxa recovered from the Dayingzhuang phytoliths include morphotypes belonging to the Pooidae subfamily, attested in samples 30–26 (period 1); and the Chloroideae, Panicoideae, Bambusoideae subfamilies, which are however present in generally lower inputs (Fig. 19); in addition, a few examples of the grass tibre Tritiaceae and the family Cyperaceae were noted.

#### Rice cultivation ecology: inferences from phytoliths

Madella et al. (2009) and Jenkins et al. (2011) outlined a methodology using the ratio of fixed vs. sensitive grass phytolith morphotypes to investigate irrigation and water usage at archaeological sites. This methodology has successfully been applied to sites in China (i.e. Weisskopf et al. 2015), allowing discussion of crop ecology, especially rice. Fixed morphotypes include short single cells, such as rondels, saddles, bilobates, and crosses; the production of these morphotypes is genetically determined, and specific plant species produce them independently of their water intake. Sensitive morphotypes include long cells, such as long smooth, echinate, and dendritic cells. Differently than short cells, long cell silicification depends on the water intake of the plant during growth, and therefore, if found in higher quantities than fixed morphotypes, they indicate the plant grew under a wet ecological regime (Madella et al. 2009).

Phytoliths from the Dayingzhuang site were classified according the above categories as outlined in Madella et al. (2009), and Weisskopf et al. (2015). While the precise distinction between wet and dry rice in terms of this ratio may be open to some discussion, it is clear that low values indicate dry (rainfed rice). Based on recent Indian work Kingwell-Banham (2019a, b) took as a dry ecology a sensitive: fixed ratio values < 1.5, whereas Fuller et al. (2016): Fig. 5) took ratios < 1 as definitely dry, with wet ratios perhaps as low as 1.25, and semi-wet ratio between 0.9 and 1.2. Kingwell-Banham (2019a, b) also inferred that values > 3 indicate systematic irrigation, whereas intermediate values (1.5–3) are wet ecologies but not necessarily irrigated. We expect transplanted rice systems to fall in the irrigated range.

**Fig. 20:**
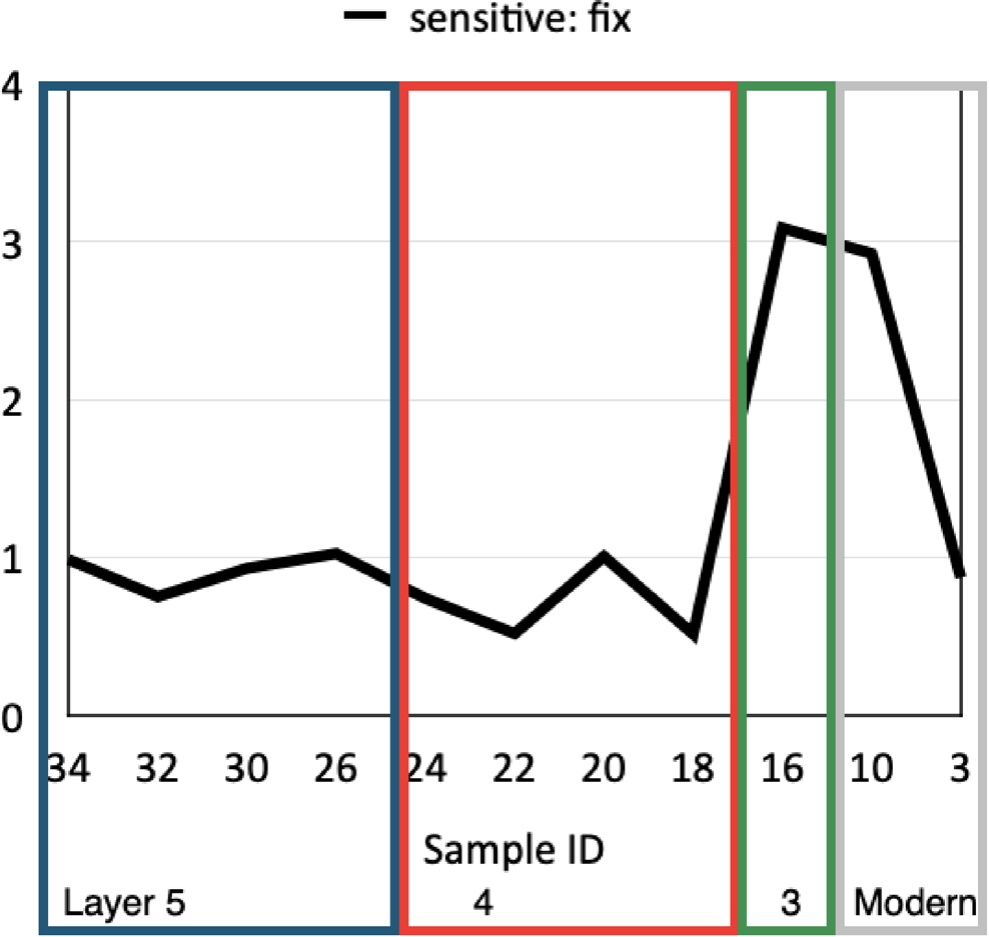
Sensitive: fixed phytolith ratio per sample at Dayingzhuang

**Fig. 21:**
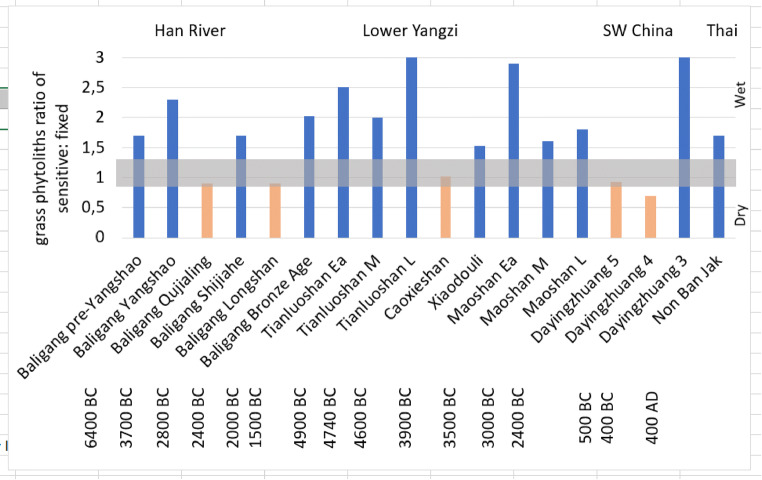
Comparison of Sensitive: Fixed values from published sites in China and Southeast Asia, data from Fuller et al. 2016

The ratios of Dayingzhuang suggest a transition from rainfed to wet and irrigated and/or transplanted rice over the course of the sites occupation (Fig. 20). Despite the high percentage of rice-derived phytoliths, some samples still showed very dry sensitive to fixed morphotype ratios (e.g. samples 20 and 18). The single sample from Layer 3 (no. 16), with a ratio of over 3, indicates a wetter ecology, possibly irrigated rice (Table 4; Figs. 20 and 21). Earlier samples also have drier S:F ratio (< 1). This suggests that earlier phases may have cultivated rice under direr conditions, based mainly on rainfall or rainy season flood recession. In general, 800 mm of rainfall is regarded as the minimal precipitation to support rainfed rice (Jacquot and Courtois 1987; Fuller et al. 2011). Nevertheless, some caution is warranted as it is possible that the samples come from multiple activities and inputs and do not only reflect plants harvested from agricultural fields; larger scale phytolith analyses across multiple contexts in each period would be needed to clarify this, but the high levels of rice phytoliths suggests that rice crop waste was a major source of phytoliths. We therefore tentatively conclude that drier field conditions closer to traditional rainfed cultivation persisted up until Layer 3, with more intensive, irrigated rice cultivation starting sometime after the fourth century BC. These phytolith data could support the inference from pollen, that more intensive and irrigated rice cultivation became widespread during the Dian period in the later 1st Millennium BC (Sun et al. 1986; Yao 2016; Xiao et al. 2020).

**Table 4:** Breakdown of *Oryza* phytoliths percentage in relation to each sample sensitive: fixed ratio per sample from Dayingzhuang. From Dal Martello 2020

Layer	Sample ID	*Oryza* morphotypes %	S:F ratio
Modern	3	2.72	0.87
	10	6.35	2.92
3	16	28.45	3.08
4	18	39.54	0.51
	20	53.71	1
	22	7.09	0.51
	24	20.19	0.74
5	26	9.96	1.02
	30	9.15	0.93
	32	7.03	0.75
	34	4.51	0.98

## Discussion: characterizing Dian agriculture

Current archaeobotanical studies in Yunnan have shown how agricultural practices in the region started from at least the mid-3rd millennium BC (Dal Martello et al. 2018; Dal Martello 2020). At the site of Baiyangcun, remains of morphologically domesticated rice and Chinese millets (*Setaria italica, Panicum miliaceum*) have been found together dating from about 2650 cal BC. The mixed rice-millet farming at Baiyangcun persisted until ca. 1600 BC and the two crops have also been found together at the slightly later site of Dadunzi, dating to AMS 2140–1630 cal BC, also located in Northwest Yunnan. At present, the archaeobotanical evidence points to a millets-rice mixed crop agriculture that came into the province from the north as the earliest form of agriculture in the region. This type of agriculture is well suited to the vertical zonation of landscape present in Yunnan. The vertical zonation coupled with the rich hydrological network allows for a variety of environmental niches to co-exist within short distances, with well-watered rice in the lower alluvial plains, and rainfed millet further away from water and surrounding slopes.

Remains of wheat and barley in Yunnan have been found at sites dating to at least a millennium later than the first appearance of agricultural crops in the province. Both wheat and barley have been reported from the second period of occupation at Haimenkou, in the Jinsha basin, Northwest Yunnan. Here, the crops have been directly dated through AMS radiocarbon dating to ca. 1400 BC (Xue 2010; Jin 2013; Dal Martello 2020). Wheat has also been reported from the Dian site of Shangxihe, in central Yunnan; this site, although culturally associated with the Dian, has been dated to c. 1200–200 BC (Yao et al. 2020; YPICRA et al. 2019), which predates the traditional beginning of the Dian by several hundred years, and wheat grains have been directly dated to 1095–933 BC at the earliest occurrence (Yao et al. 2020). Instead, almost all of the sites dating to the 1st millennium BC have reported wheat and barley remains, indicating that the crops had dispersed widely in the province by the 1st millennium BC. These include the Dian sites of Shangxihe, Shizhaishan, Hebosuo, Anjiang, Xueshan, Guangfentou, and Xiaogucheng, with the exclusion of Shangxihe, all roughly dating from the eighth century BC onward (i.e. Wang 2014; Yang 2016; Li and Liu 2016; Yao et al. 2015).

There are still debates on the route and timing through which wheat and barley reached Yunnan, with earlier evidence in both South Asia and Central Asia (Fig. 11). One hypothesis is that they spread together through the southward dispersal of populations from Northwest China during the Bronze Age. This is supported by changes in material culture and general subsistence composition, such as at Haimenkou where an increase of sheep/goat remains took place alongside the appearance of wheat and barley, present by ca. 1400 BC (e.g. Xue 2010; Stevens et al. 2016; Dal Martello 2020). This period also indicated increased population at the site, with a rise in houses and tree-cutting tools. However, in much northern and central China early wheat occurred in the absence of evidence for barley so it may not be the case that these crops spread together (e.g. Flad et al. 2010; Boivin et al. 2012; Liu et al. 2017; Deng et al. 2020). Wheat and barley co-occur the site of A’shaonao, on the eastern edge of the Tibetan Plateau in Sichuan, where the crops have been directly dated to c. 1400 BC (d'Alpoim Guedes et al. 2015), contemporary to the finds of Haimenkou.

An alternative hypothesis posits that the arrival of barley in China came from the southwest (from India) separately from the arrival of wheat from Central Asia to Northwest China at the end of the Neolithic (e.g. Lister et al. 2018). Currently, there is limited evidence of the arrival of barley, and available dating evidence for neither route is conclusive. When present in Yunnan, barley appears as minor and always co-occurs with more substantial quantities of wheat. Recently obtained direct radiocarbon dates on barley grains recovered from interior sites on the northeastern Tibetan Plateau are earlier than those from either Xinjiang (northwest) and southern Tibet (southwest). At the site of Xiasunjiazhai, on the northeastern side of the Tibetan Plateau in Qinghai province, barley grains have been dated to 2136–1959 cal BC (Liu et al. 2017), whereas at the sites of Sidaogou and Yanghai, in Xinjiang, barley dates to 978–831 cal BC and 750–405 cal BC respectively, and at Khog gzung and Bangtangbu, on the south-western Tibetan Plateau, it dates to 1393–1211 cal BC, and 1263–1056 cal BC respectively (Liu et al. 2017; Lister et al. 2018), which is similary to both dates from Haimenkou and A’shaonao. Outside China, evidence relating a posited northwestern/Central Asia source include barley finds from Ojakly in Turkmenistan 1617–1498 cal BC, Tasbas in Kazakhstan 1437–1233 cal BC; and Aigyrchal-2 in Kyrgyzstan 1630–1497 cal BC (Spengler 2015; Lister et al. 2018); these are not earlier than Xiansunjiazhai and again comparable to dates from Haimenkou and A’shaonao. Barley finds that might relate to precursors on the Southwestern route, include grains from Kanispur in Kashmir directly dated to 2467–2236 cal BC (Liu et al. 2017), or evidence from the Lower Ganges valley at Chirand in Bihar, where barley and rice are present with associated dates from 1920 to 1660 BC (Vishnu-Mittre 1972; Fuller 2011). In Nepal, the only early archaeobotanical evidence places barley in the eastern Himalayas, at the site of Chokhopani, from ca. 1000 BC (Knörzer 2000). Currently, the route or routes by which barley arrived in Yunnan are unclear, as well as whether this took place with wheat or other cultural elements. Genetic data from goats suggests a dispersal took place from South Asia to Southeast and Southwest China (mt-DNA haplogroup B) separately from a dispersal from Central Asia into China (haplogroup A; see Lin et al. 2013; Waki et al. 2015). In contrast, Chinese sheep, like wheat, appear to have arrived from Central Asia via the Northwest and/or Mongolia (Sun et al. 2010). Further archaeological evidence is needed to resolve when domesticates arrived from the Southwest into China as distinct from those that arrived via the Northwest.

**Fig. 22:**
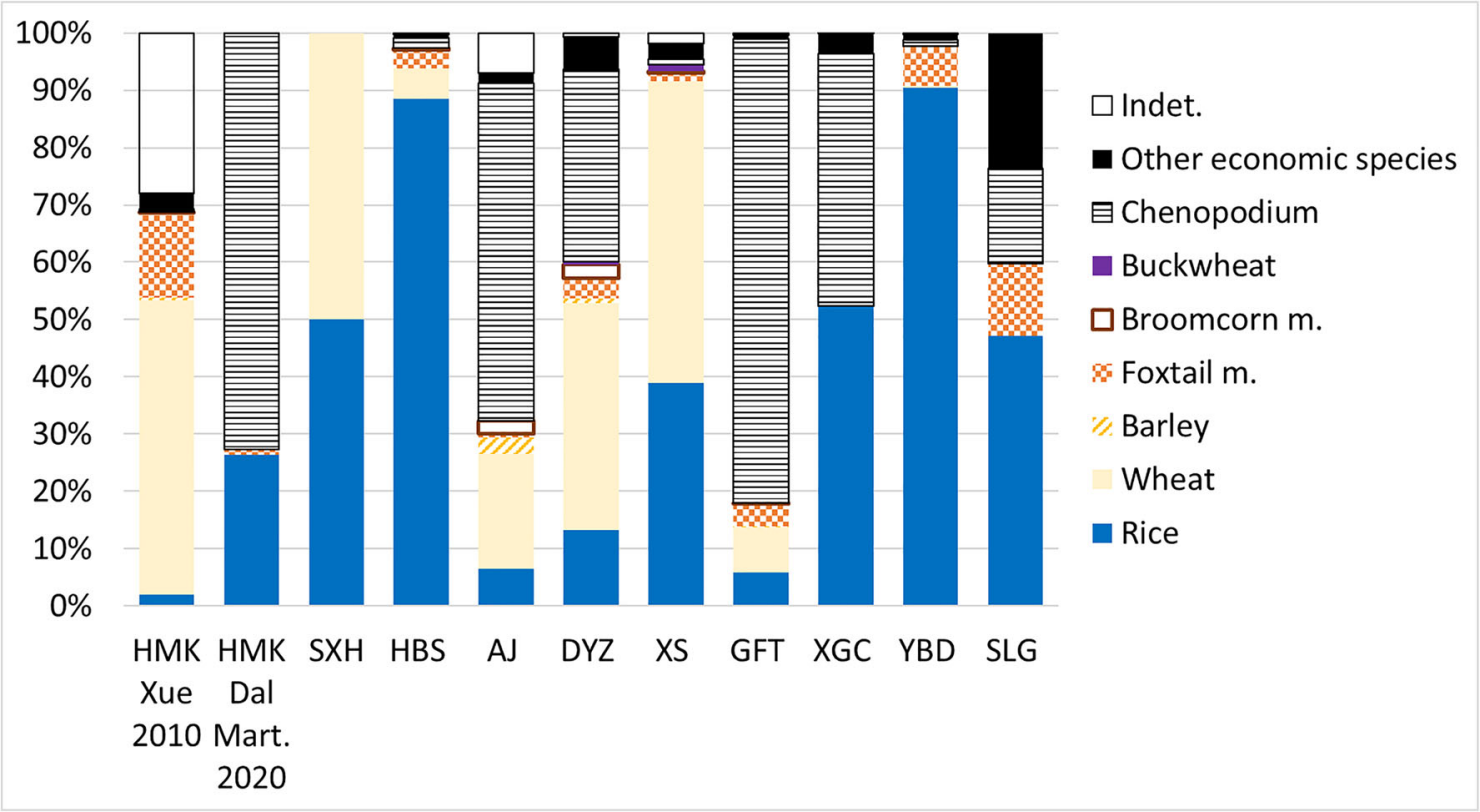
Comparison of frequency index for sites in Yunnan with flotation, dating between c. 1000-300 BC. The site initials are used to indicate the site (HMK= Haimenkou; SLG= Shilingang; YBD= Yubeidi; HBS= Hebosuo; AJ= Anjiang; DYZ= Dayingzhuang; XS= Xueshan; GFT= Guangfentou; XGC= Xiaogucheng) .HMK=Haimenkou refers to the 3rd period of occupation of the site dated to between 800-400 BC. Data from: Dal Martello 2020; Xue 2010; Jin 2013; Yao et al. 2015; Li et al. 2016; Wang 2014; Li and Liu 2016). Remade from Dal Martello 2020

The recent archaeobotanical studies carried out at the sites within the Dian sphere of influence (including sites surrounding Lakes Dian, Fuxian, and Xingyun) illustrate what can be described as the “Dian agricultural system”. This is characterised by a high mixture of crops, including seasonally differentiated crops, such as summer rice and millets, both foxtail and broomcorn millet, and winter wheat and barley (Fig. 22). The specific ratio of these species varies among each site; however, rice and wheat seem to be the two most prevalent species found across all sites (Fig. 22). Additionally, *Chenopodium* remains have also been reported in considerable quantities, especially at the site of Guangfentou (Li and Liu 2016). *Chenopodium* can be exploited as a food resource, and historically it has been a minor crop, and trade item, in parts of the Himalayas and Tibet up to the present day, including being cultivated for both its seeds and leaves among the Formosan tribes in highland Taiwan. Few scholars have proposed that this species might have been exploited as food at other nearby Chinese earlier sites of Haimenkou (Xue 2010; Dal Martello) in northwestern Yunnan and Guiyuanqiao in Sichuan (d’Alpoim et al. 2015); however, there has been no systematic exploration of this issue in the context of Dian Yunnan. The consistent finding of a high quantity of *Chenopodium* from early sites in Yunnan deserves future scholarly attention and archaeobotanical investigation. Finally, the archaeobotanical record shows only a limited input from wild resources. Nevertheless, some intensive use of wild aquatic animal resources was likely. Sites like Dayingzhuang, which is a shell-midden, included numerous lacustrine remains (fish and shellfish). No in depth zooarchaeological analyses have been published from this period from which to address the role of pigs, which were certainly present in the region since the Neolithic (Wang 2018), or whether any domesticated bovids were yet present.

It is posited that general weather conditions in the Dian Basin during the 1st millennium were largely comparable to those of present day, albeit fluctuating. Today, modern agriculture is characterised by multiple crops per year, with a harvest of summer rice and a harvest of winter wheat (Zhao 1986). The fluctuating climate might have had a role in encouraging the preservation of this highly mixed crop system.

## Conclusion

The variety of crops recovered from all of these sites suggest that already during the Dian, people were taking advantage of the vertical landscape and multiple seasons of agriculture to maximise production. Wet rice fields are likely to have been concentrated at the bottom of the valley, with cultivation of rainfed crops on the off seasons (winter) or in the surrounding hills (summer millet). These two seasons of agriculture represent intensification of land use since the Neolithic when cropping was only focused on the summer. Further intensification of summer rice could have taken place through artificial irrigation to maintain higher water levels and transplanting. At present, it is still difficult to establish when this happened, although its presence in the Han period is generally agreed, and the high sensitive: fixed phytolith ratio from Layer 3 would support its presence at Dayingzhuang before the site was abandoned around the fourth century BC.

## Supplementary information

ESM 1 (DOCX 16 kb)

ESM 2 (XLSX 19 kb)

ESM 3 (DOCX 45 kb)

ESM 4 (DOCX 83 kb)

ESM 5 (XLSX 13 kb)

## Data Availability

Data are included as supplementary files to this publication. Flotation samples are stored with the Yunnan Province Institute of Archaeology and Cultural Relics. Sorted, photographed, and measured seeds as well as phytolith slides are currently in the archaeobotany laboratory at UCL and due to be returned to Yunnan for long-term archiving.

## Footnotes

1 In trench TN2E2, a total of 5 multiple samples for each individual layer were collected, one at each corner of the trench (S1–4), and one (S5) in the middle, respectively. These were individually sorted, and the results from multiple samples from the same layer were merged before performing quantitative analyses.

2 Originally, sample #12 (from layer 3) was also processed to slide, but after mounting to slide was revealed to be too badly mounted to be successfully analysed and was thus discarded,
