# Supplementary material for: Two-season agriculture and irrigated rice during the Dian: radiocarbon dates and archaeobotanical remains from Dayingzhuang, Yunnan, Southwest China
Source: Archaeol Anthropol Sci. 2021 Mar 13;13(4):62. doi: 10.1007/s12520-020-01268-y (PMC7956011; doi:10.1007/s12520-020-01268-y)
Supplement: Supplementary file 1 — (DOCX 16 kb) [file 12520_2020_1268_MOESM1_ESM.docx]

**S1. Phytoliths laboratory processing protocols**

Laboratory extraction was carried out at the UCL Institute of Archaeology-Phytoliths Laboratory following an adaptation of the A. M. Rosen (1999) protocol as outlined below.

Day 1:

1. Soil from each sample was ground using a marble pestle and mortar, and then sifted through a 0.25mm sieve.

Pestle, mortar, and sieve were systematically cleaned with soapy warm water in between each sample to avoid contamination.

About 1.2g of sieved soil was weighted and poured into a plastic tube.

Tubes were labelled 1-12. Weights were recorded.

1. 15ml of 10% HCL were added to each sample and tubes were shacked gently in a fume cupboard.
2. About 40ml of deionised water were added to each tube to be balanced on scales in pair and put in the centrifuge.

Tubes were centrifuged for 5 minutes at 2000rpm. The excess water was then poured off, and this whole step was repeated a total of 3 times.

1. About 5ml of distilled water were added to each sample, and they were let to sit overnight.

Day 2:

1. The excess water was pipetted out from each plastic tube.
2. About 20ml of 10% calgon solution was added, and each tube shaked.

The content was then transferred to glass beakers, washing out the tubes with deionised water to transfer anything residue left in the tube.

1. Each beaker was filled up to 8cm height with deionised water and stirred gently, then let to sit first for 1hour and 10minutes.

The top layer of water was then poured off and more water was added up to the 8cm mark. After stirring again, the samples were let to sit for 1 hour exactly, and the whole process was repeated until the water was clear.

1. The top layer of clear suspense was poured off, and each sample was transferred into a crucible using a pipette.
2. Samples on crucibles were dried in the drying oven at 50 ºC overnight.

Day 3:

1. After taking the samples out of the oven, they were each broken up and put in the muffle furnace at 500°C for 2 hours.
2. At the end of the 2 hours, samples were taken out of the furnace and let to cool.
3. Each sample was scraped from the crucible and poured into a 15ml tube with the aid of a piece of shiny plastic paper.
4. 3ml of sodium polytungstate solution were added to each tube, which was then shaked.
5. About 12 ml of deionised water were added to the samples, which were balanced on scales in pairs, and then centrifuged for 10 minutes at 800rpm.
6. The suspense (containing the phytolith) was poured in a new 15ml tube, to which more deionised water was added.
7. The samples were centrifuged three times for 5 minutes at 2000 rpm, pouring away the suspense and re-adding deionised water in between as outlined in steps 14-15 above.
8. After the last centrifuge, the suspense was poured away, and the phytoliths (now at the bottom) were transferred to small glass beakers with a pipette. Weights were recorded.
9. The beakers were finally put in the drying over overnight.

Day 4:

1. Dry phytoliths and pot weights were recorded.
2. About 2.5mg of phytoliths from each sample was weighted and mounted on a glass slide using Entellan. After spreading Entellan on a premeasured square on the glass slide, a toothpick was used to spread the phytoliths evenly across the square. A thin glass square cover was then put over it, and the slides were put horizontally to dry.

Each slide was labelled with sample ID and mounted phytoliths weight (see fig. 4-8).

1. The rest of the phytoliths were put in individual plastic tubes for storage.
