# Supplementary material for: Two-season agriculture and irrigated rice during the Dian: radiocarbon dates and archaeobotanical remains from Dayingzhuang, Yunnan, Southwest China
Source: Archaeol Anthropol Sci. 2021 Mar 13;13(4):62. doi: 10.1007/s12520-020-01268-y (PMC7956011; doi:10.1007/s12520-020-01268-y)
Supplement: Supplementary file 3 — (DOCX 45 kb) [file 12520_2020_1268_MOESM3_ESM.docx]

**Supplementary file 3.** Grain Measurements on main species from Dayingzhuang.

| **DAYINGZHUANG** | | | | ***Oryza sativa*** | | | |
| --- | --- | --- | --- | --- | --- | --- | --- |
| **No.** | **Site** | **Context** | **Period** | **Length** | **Width** | **Thickness** | **L/W** |
| 1 | 2017 YHD | H8 | 2 | 5.09 | 2.13 | 2.09 | 2.38967136 |
| 2 | 2017 YHD | H8 | 2 | 4.72 | 2.26 | 1.36 | 2.08849558 |
| 3 | 2017 YHD | H8 | 2 | 4.79 | 2.66 | 2.62 | 1.80075188 |
| 4 | 2017 YHD | H8 | 2 | 5.02 | 2.85 | 2.4 | 1.76140351 |
| 5 | 2017 YHD | H8 | 2 | 3.1 | 2.29 | 2.6 | 1.35371179 |
| 6 | 2017 YHD | Layer 2 | 3 | 4.86 | 2.77 | 2.16 | 1.75451264 |
| 7 | 2017 YHD | Layer 2 | 3 | 5.12 | 2.81 | 2.4 | 1.82206406 |
| 8 | 2017 YHD | Layer 3 | 3 | 5.35 | 2.96 | 2.26 | 1.80743243 |
| 9 | 2017 YHD | Layer 5 | 1 | 5.33 | 3.07 | 2.72 | 1.73615635 |
| 10 | 2017 YHD | Layer 5 | 1 | 4.57 | 2.36 | 1.66 | 1.93644068 |
| 11 | 2017 YHD | Layer 5 | 1 | 4.57 | 2.96 | 2.33 | 1.54391892 |
| 12 | 2017 YHD | Layer 5 | 1 | 4.69 | 2.71 | 1.91 | 1.73062731 |
| 13 | 2017 YHD | Layer 5 | 1 | 4.37 | 2.77 | 2.15 | 1.57761733 |
| 14 | 2017 YHD | H30 | 1 | 4.06 | 2.7 | 1.92 | 1.5037037 |
| 15 | 2017 YHD | H34 | 1 | 4.43 | 2.53 | 1.78 | 1.75098814 |
| 16 | 2017 YHD | H11 | 2 | 5.24 | 2.86 | 2.33 | 1.83216783 |
| 17 | 2017 YHD | H28 | 1 | 4.52 | 2.9 | 2.24 | 1.55862069 |
| 18 | 2017 YHD | H28 | 1 | 4.36 | 2.61 | 1.72 | 1.67049808 |
| 19 | 2017 YHD | H8 | 2 | 4.65 | 2.88 | 2.17 | 1.61458333 |
| 20 | 2017 YHD | H8 | 2 | 5.49 | 2.46 | 2.16 | 2.23170732 |
| 21 | 2017 YHD | Jicao 4 | 1 | 4.68 | 2.27 | 1.93 | 2.06167401 |
| **DAYINGZHUANG**  ***Oryza sativa*** | | | **AVERAGE** | **4.7147** | **2.6576** | **2.1385** | **1.78698795** |
|  |  |  | **STDEV** | **0.5259** | **0.2702** | **0.3394** | **0.24785926** |

| **DAYINGZHUANG** | | | | ***Setaria italica*** | | | |
| --- | --- | --- | --- | --- | --- | --- | --- |
| **No.** | **Site** | **Context** | **Period** | **Length** | **Width** | **Thickness** | **L/W** |
| 1 | 2017 YHD | H9 | 2 | 1.4 | 1.14 | 1.13 | 1.22807018 |
| 2 | 2017 YHD | H9 | 2 | 1.12 | 1.33 | 1 | 0.84210526 |
| 3 | 2017 YHD | H9 | 2 | 1.2 | 1.32 | 1.03 | 0.90909091 |
| 4 | 2017 YHD | 5 s3 | 1 | 1.29 | 1.21 | 1.05 | 1.0661157 |
| 5 | 2017 YHD | 5 s3 | 1 | 0.98 | 1.04 | 0.76 | 0.94230769 |
| 6 | 2017 YHD | F1 | 3 | 0.87 | 1 | 0.87 | 0.87 |
| 7 | 2017 YHD | H30 | 1 | 1.2 | 1.22 | 0.86 | 0.98360656 |
| 8 | 2017 YHD | H30 | 1 | 1.2 | 1.39 | 1.04 | 0.86330935 |
| 9 | 2017 YHD | H28 | 1 | 1.18 | 1.28 | 1.11 | 0.921875 |
| 10 | 2017 YHD | H33 | 1 | 1.33 | 1.33 | 0.97 | 1 |
| 11 | 2017 YHD | H33 | 1 | 1.09 | 1.06 | 0.84 | 1.02830189 |
| 12 | 2017 YHD | H8 | 2 | 1.15 | 1.18 | 0.98 | 0.97457627 |
| 13 | 2017 YHD | H8 | 2 | 1.33 | 1.29 | 0.91 | 1.03100775 |
| 14 | 2017 YHD | H8 | 2 | 0.93 | 1.16 | 0.88 | 0.80172414 |
| 15 | 2017 YHD | H8 | 2 | 1.13 | 1.16 | 0.95 | 0.97413793 |
| 16 | 2017 YHD | H8 | 2 | 1.2 | 1.29 | 1.06 | 0.93023256 |
| 17 | 2017 YHD | H8 | 2 | 1.18 | 1.11 | 0.98 | 1.06306306 |
| 18 | 2017 YHD | H8 | 2 | 1.11 | 1.06 | 0.84 | 1.04716981 |
| **DAYINGZHUANG**  ***Setaria italica*** | | | **AVERAGE** | **1.1605** | **1.1983** | **0.9588** | **0.9709274** |
|  |  |  | **STDEV** | **0.1369** | **0.1156** | **0.1026** | **0.1009117** |

| **DAYINGZHUANG** | | | | ***Panicum miliaceum*** | | | |
| --- | --- | --- | --- | --- | --- | --- | --- |
| **No.** | **Site** | **Context** | **Period** | **Length** | **Width** | **Thickness** | **L/W** |
| 1 | 2017 YHD | Hedao1-2 | 1 | 1.88 | 1.71 | 1.17 | 1.0994152 |
| 2 | 2017 YHD | Hedao1-2 | 1 | 1.59 | 1.49 | 1.15 | 1.06711409 |
| 3 | 2017 YHD | Hedao1-2 | 1 | 1.73 | 1.83 | 1.33 | 0.94535519 |
| 4 | 2017 YHD | Hedao 1 | 1 | 1.57 | 1.57 | 1.31 | 1 |
| 5 | 2017 YHD | Hedao 1 | 1 | 1.86 | 1.54 | 1.16 | 1.20779221 |
| 6 | 2017 YHD | Hedao 1 | 1 | 1.7 | 1.75 | 1.34 | 0.97142857 |
| 7 | 2017 YHD | Hedao 1 | 1 | 1.72 | 2.03 | 1.13 | 0.84729064 |
| 8 | 2017 YHD | Hedao 1 | 1 | 1.76 | 1.69 | 1.46 | 1.04142012 |
| 9 | 2017 YHD | Hedao 1 | 1 | 1.67 | 1.76 | 1.28 | 0.94886364 |
| 10 | 2017 YHD | Hedao 1 | 1 | 1.78 | 1.52 | 0.89 | 1.17105263 |
| 11 | 2017 YHD | H30 | 1 | 1.93 | 2.12 | 1.52 | 0.91037736 |
| 12 | 2017 YHD | H15 | 2 | 2.01 | 2.16 | 1.6 | 0.93055556 |
| 13 | 2017 YHD | H28 | 1 | 1.93 | 1.6 | 1.64 | 1.20625 |
| 14 | 2017 YHD | H29 | 1 | 1.47 | 1.58 | 1.44 | 0.93037975 |
| 15 | 2017 YHD | H29 | 1 | 1.85 | 1.68 | 1.64 | 1.10119048 |
| **DAYINGZHUANG**  ***Panicum miliaceum*** | | | **AVERAGE** | **1.7633** | **1.7353** | **1.3373** | **1.02523236** |

| **DAYINGZHUANG** | | | | ***Triticum aestivum*** | | | |
| --- | --- | --- | --- | --- | --- | --- | --- |
| **No.** | **Site** | **Context** | **Period** | **Length** | **Width** | **thickness** | **L/W** |
| 1 | 2017 YHD | H9 | 2 | 3.39 | 2.55 | 2.05 | 1.32941176 |
| 2 | 2017 YHD | HDM3 | 2 | 4.12 | 2.46 | 2.39 | 1.67479675 |
| 3 | 2017 YHD | HDM3 | 2 | 4.32 | 2.83 | 2.54 | 1.52650177 |
| 4 | 2017 YHD | HDM3 | 2 | 3.55 | 2.27 | 2.16 | 1.56387665 |
| 5 | 2017 YHD | Hedao 1 | 1 | 3.37 | 2.75 | 2.39 | 1.22545455 |
| 6 | 2017 YHD | Hedao 1 | 1 | 4.81 | 3.55 | 2.99 | 1.35492958 |
| 7 | 2017 YHD | Hedao 1 | 1 | 4.15 | 3.18 | 2.77 | 1.30503145 |
| 8 | 2017 YHD | Hedao 1 | 1 | 3.91 | 3.3 | 2.95 | 1.18484848 |
| 9 | 2017 YHD | Hedao 1 | 1 | 3.48 | 2.77 | 2.24 | 1.25631769 |
| 10 | 2017 YHD | Hedao 1 | 1 | 4.17 | 3.09 | 2.99 | 1.34951456 |
| 11 | 2017 YHD | Hedao 1 | 1 | 3.97 | 3.16 | 2.94 | 1.25632911 |
| 12 | 2017 YHD | Hedao 1 | 1 | 4.44 | 3.49 | 2.88 | 1.2722063 |
| 13 | 2017 YHD | Hedao 1 | 1 | 4.55 | 2.9 | 2.27 | 1.56896552 |
| 14 | 2017 YHD | Hedao 1 | 1 | 4.6 | 3.2 | 2.86 | 1.4375 |
| 15 | 2017 YHD | Hedao 1 | 1 | 4.27 | 3.09 | 2.77 | 1.38187702 |
| 16 | 2017 YHD | Hedao 1 | 1 | 3.66 | 2.78 | 2.42 | 1.31654676 |
| 17 | 2017 YHD | Hedao 1 | 1 | 3.79 | 3.09 | 2.64 | 1.22653722 |
| 18 | 2017 YHD | Hedao 1 | 1 | 3.89 | 2.87 | 2.62 | 1.3554007 |
| 19 | 2017 YHD | Hedao 1 | 1 | 3.84 | 2.76 | 2.41 | 1.39130435 |
| 20 | 2017 YHD | Hedao 1 | 1 | 3.86 | 2.99 | 2.7 | 1.2909699 |
| 21 | 2017 YHD | Hedao 1 | 1 | 4.23 | 2.89 | 2.27 | 1.46366782 |
| 22 | 2017 YHD | Hedao 1 | 1 | 3.78 | 3.05 | 2.88 | 1.23934426 |
| 23 | 2017 YHD | Hedao 1 | 1 | 4.32 | 2.74 | 2.48 | 1.57664234 |
| 24 | 2017 YHD | Hedao 1 | 1 | 3.78 | 2.86 | 2.5 | 1.32167832 |
| 25 | 2017 YHD | Hedao 1 | 1 | 4.5 | 3.31 | 2.8 | 1.35951662 |
| 26 | 2017 YHD | Hedao 1 | 1 | 4.93 | 3.44 | 2.69 | 1.43313953 |
| 27 | 2017 YHD | Hedao 1 | 1 | 4.14 | 2.55 | 2.42 | 1.62352941 |
| 28 | 2017 YHD | Hedao 1 | 1 | 4.29 | 3.08 | 2.45 | 1.39285714 |
| 29 | 2017 YHD | Hedao 1 | 1 | 3.75 | 2.46 | 2.56 | 1.52439024 |
| 30 | 2017 YHD | Hedao 1 | 1 | 3.46 | 2.76 | 2.36 | 1.25362319 |
| 31 | 2017 YHD | Hedao 1 | 1 | 3.62 | 2.83 |  | 1.27915194 |
| 32 | 2017 YHD | Hedao 1 | 1 | 4.01 | 3.01 |  | 1.33222591 |
| 33 | 2017 YHD | Hedao 1 | 1 | 3.71 | 2.97 |  | 1.24915825 |
| 34 | 2017 YHD | Hedao 1 | 1 | 4.89 | 3.3 |  | 1.48181818 |
| 35 | 2017 YHD | Hedao 1-2 | 1 | 4.69 | 3.11 | 2.68 | 1.50803859 |
| 36 | 2017 YHD | Hedao 1-2 | 1 | 4.53 | 3.81 | 2.74 | 1.18897638 |
| 37 | 2017 YHD | Hedao 1-2 | 1 | 3.55 | 3 | 2.3 | 1.18333333 |
| 38 | 2017 YHD | Hedao 1-2 | 1 | 4.25 | 3.03 | 2.61 | 1.40264026 |
| 39 | 2017 YHD | Hedao 1-2 | 1 | 4.12 | 2.94 | 2.77 | 1.40136054 |
| **DAYINGZHUANG** | | | | ***Triticum aestivum*** | | | |
| **No.** | **Site** | **Context** | **Period** | **Length** | **Width** | **thickness** | **L/W** |
| 40 | 2017 YHD | Hedao 1-2 | 1 | 4.49 | 3.3 | 2.63 | 1.36060606 |
| 41 | 2017 YHD | Hedao 1-2 | 1 | 3.77 | 2.97 | 2.5 | 1.26936027 |
| 42 | 2017 YHD | Hedao 1-2 | 1 | 4.01 | 3.16 | 2.53 | 1.26898734 |
| 43 | 2017 YHD | Hedao 1-2 | 1 | 4.3 | 2.99 | 2.73 | 1.43812709 |
| 44 | 2017 YHD | Hedao 1-2 | 1 | 4.12 | 2.82 | 2.61 | 1.46099291 |
| 45 | 2017 YHD | Hedao 1-2 | 1 | 4.04 | 2.93 | 2.81 | 1.37883959 |
| 46 | 2017 YHD | Hedao 1-2 | 1 | 4.18 | 2.97 | 2.67 | 1.40740741 |
| 47 | 2017 YHD | Hedao 1-2 | 1 | 4.34 | 3.56 | 3.02 | 1.21910112 |
| 48 | 2017 YHD | Hedao 1-2 | 1 | 5.03 | 3.4 | 3.06 | 1.47941176 |
| 49 | 2017 YHD | Hedao 1-2 | 1 | 4.08 | 3.1 | 2.42 | 1.31612903 |
| 50 | 2017 YHD | Hedao 1-2 | 1 | 4.04 | 3.1 | 2.59 | 1.30322581 |
| 51 | 2017 YHD | Hedao 1-2 | 1 | 4.4 | 3.54 | 2.83 | 1.24293785 |
| 52 | 2017 YHD | Hedao 1-2 | 1 | 4 | 3.49 | 2.67 | 1.14613181 |
| 53 | 2017 YHD | Hedao 1-2 | 1 | 3.91 | 3.05 | 2.2 | 1.28196721 |
| 54 | 2017 YHD | Hedao 1-2 | 1 | 4.25 | 3.27 | 2.72 | 1.29969419 |
| 55 | 2017 YHD | Hedao 1-2 | 1 | 3.72 | 2.91 | 1.99 | 1.27835052 |
| 56 | 2017 YHD | Hedao 1-2 | 1 | 4.51 | 3.46 | 2.61 | 1.30346821 |
| 57 | 2017 YHD | Hedao 1-2 | 1 | 4.36 | 2.87 | 2.54 | 1.51916376 |
| 58 | 2017 YHD | Hedao 1-2 | 1 | 4.08 | 2.82 | 2.64 | 1.44680851 |
| 59 | 2017 YHD | Hedao 1-2 | 1 | 3.7 | 2.62 | 2.24 | 1.41221374 |
| 60 | 2017 YHD | Hedao 1-2 | 1 | 4.14 | 2.66 | 2.53 | 1.55639098 |
| 61 | 2017 YHD | Hedao 1-2 | 1 | 4.85 | 3.45 | 2.97 | 1.4057971 |
| 62 | 2017 YHD | Hedao 1-2 | 1 | 3.81 | 2.98 | 2.6 | 1.27852349 |
| 63 | 2017 YHD | Hedao 1-2 | 1 | 4.32 | 3.49 | 2.67 | 1.23782235 |
| 64 | 2017 YHD | Hedao 1-2 | 1 | 4.54 | 3.31 | 3.17 | 1.37160121 |
| 65 | 2017 YHD | Jicao 2 | 1 | 4.62 | 3.02 | 2.39 | 1.52980132 |
| 66 | 2017 YHD | Jicao 2 | 1 | 4.34 | 2.07 | 1.67 | 2.09661836 |
| 67 | 2017 YHD | Layer 2 | 3 | 4.71 | 3.57 | 2.31 | 1.31932773 |
| 68 | 2017 YHD | Layer 3 | 3 | 3.16 | 2.24 | 2.13 | 1.41071429 |
| 69 | 2017 YHD | Layer 3 | 3 | 3.57 | 2.95 | 2.59 | 1.21016949 |
| 70 | 2017 YHD | Layer 3 | 3 | 3.9 | 2.84 | 2.48 | 1.37323944 |
| 71 | 2017 YHD | Layer 3 | 3 | 3.95 | 2.81 | 2.63 | 1.40569395 |
| 72 | 2017 YHD | Layer 4 | 2 | 4.43 | 2.76 | 2.42 | 1.60507246 |
| 73 | 2017 YHD | Layer 4 | 2 | 4.44 | 3.09 | 2.92 | 1.4368932 |
| 74 | 2017 YHD | Layer 4 | 2 | 3.58 | 2.22 | 2.08 | 1.61261261 |
| 75 | 2017 YHD | Layer 5 | 1 | 3.75 | 2.57 | 2.24 | 1.45914397 |
| 76 | 2017 YHD | Layer 5 | 1 | 4.15 | 2.9 | 2.49 | 1.43103448 |
| 77 | 2017 YHD | Layer 5 | 1 | 3.04 | 2.12 | 2.15 | 1.43396226 |
| 78 | 2017 YHD | Layer 5 | 1 | 3.56 | 2.69 |  | 1.32342007 |
| 79 | 2017 YHD | Layer 5 | 1 | 3.24 | 2.12 |  | 1.52830189 |
| **DAYINGZHUANG** | | | | ***Triticum aestivum*** | | | |
| **No.** | **Site** | **Context** | **Period** | **Length** | **Width** | **thickness** | **L/W** |
| 80 | 2017 YHD | Layer 5 | 1 | 3.61 | 2.73 |  | 1.32234432 |
| 81 | 2017 YHD | F1 | 2 | 3.67 | 2.55 | 2.13 | 1.43921569 |
| 82 | 2017 YHD | F1 | 2 | 3.55 | 2.42 | 2.39 | 1.46694215 |
| 83 | 2017 YHD | H11 | 2 | 4.4 | 4.2 | 2.85 | 1.04761905 |
| 84 | 2017 YHD | H11 | 2 | 4.89 | 3.4 | 2.04 | 1.43823529 |
| 85 | 2017 YHD | H11 | 2 | 3.72 | 3.03 | 2.75 | 1.22772277 |
| 86 | 2017 YHD | H15 | 2 | 4.08 | 2.81 | 1.6 | 1.4519573 |
| 87 | 2017 YHD | H15 | 2 | 4.13 | 2.94 | 2.59 | 1.4047619 |
| 88 | 2017 YHD | H15 | 2 | 3.55 | 2.44 |  | 1.45491803 |
| 89 | 2017 YHD | H15 | 2 | 3.55 | 2.57 |  | 1.38132296 |
| 90 | 2017 YHD | H8 | 2 | 4.34 | 2.68 | 2.33 | 1.61940299 |
| **DAYINGZHUANG**  ***Triticum aestivum*** | | | **AVERAGE** | **4.0623** | **2.9575** | **2.5385** | **1.38441022** |
|  |  |  | **STDEV** | **0.4320** | **0.3845** | **0.3019** | **0.14378873** |

| **DAYINGZHUANG** | | | | ***Hordeum vulgare*** | | | |
| --- | --- | --- | --- | --- | --- | --- | --- |
| **No.** | **Site** | **Context** | **Period** | **Length** | **Width** | **Thickness** | **L/W** |
| 1 | 2017 YHD | Hedao 1 | 1 | 5.93 | 2.62 | 2.1 | 2.26335878 |
| 2 | 2017 YHD | Hedao 1 | 1 | 5.5 | 3.02 | 2.55 | 1.82119205 |
| 3 | 2017 YHD | Layer 4 | 2 | 4.97 | 2.5 | 2.08 | 1.988 |
| 4 | 2017 YHD | F1 |  | 4.12 | 2.3 | 1.78 | 1.79130435 |
| **DAYINGZHUANG**  ***Hordeum vulgare*** | | | **AVERAGE** | **5.13** | **2.61** | **2.1275** | **1.96596379** |
|  |  |  | **stdev** | **0.7794** | **0.3035** | **0.3174** | **0.21632857** |

| **DAYINGZHUANG** | | | ***Chenopodium* sp.** | | | | |
| --- | --- | --- | --- | --- | --- | --- | --- |
| **No.** | **Context** | **Period** | **Length** | **Nose** | **Width** | **Thickness** | **Seed coat Thickness um** |
| 1 | F1-1 | 2/3 | 1.04 | 0.132 | 0.926 | 0.341 |  |
| 2 | F1-1 | 2/3 | 1.1 | 0.135 | 0.916 | 0.618 |  |
| 3 | F1-1 | 2/3 | 1.02 | 0.145 | 0.97 | 0.368 |  |
| 4 | F1-1 | 2/3 | 1.02 | 0.129 | 0.959 | 0.335 |  |
| 5 | F1-1 | 2/3 | 1.05 | 0.114 | 0.967 | 0.61 | 39.8840526 |
| 6 | F1-1 | 2/3 | 0.918 | 0.0823 | 0.896 | 0.499 | 30.2428485 |
| 7 | F1-1 | 2/3 | 0.966 | 0.121 | 0.88 | 0.404 | 35.7919063 |
| 8 | H11 | 2/3 | 1.27 | 0.15 | 1.16 | 0.59 |  |
| 9 | H11 | 2/3 | 1.35 | 0.165 | 1.2 | 0.662 |  |
| 10 | H11 | 2/3 | 1.15 | 0.104 | 1.09 | 0.612 |  |
| 11 | H11 | 2/3 | 1.23 | 0.179 | 1.12 | 0.617 |  |
| 12 | H11 | 2/3 | 1.23 | 0.156 | 1.18 | 0.687 |  |
| 13 | H11 | 2/3 | 1.19 | 0.117 | 1.08 | 0.62 |  |
| 14 | H11 | 2/3 | 1.28 | 0.213 | 1.38 | 0.742 |  |
| 15 | H11 | 2/3 | 1.13 | 0.145 | 1.08 | 0.509 |  |
| 16 | H9 | 2/3 | 0.978 | 0.077 | 0.962 | 0.443 |  |
| 17 | H9 | 2/3 | 1.04 | 0.157 | 0.912 | 0.548 |  |
| 18 | H9 | 2/3 | 1.07 | 0.131 | 0.922 | 0.536 | 32.6842222 |
| 19 | H9 | 2/3 | 1.14 | 0.143 | 1.04 | 0.374 | 36.1108947 |
| 20 | H9 | 2/3 | 1.04 | 0.0862 | 0.992 | 0.488 |  |
| 21 | H9 | 2/3 | 1.4 | 0.219 | 1.36 | 0.532 |  |
| 22 | H9 | 2/3 | 1.27 | 0.161 | 1.09 | 0.856 | 23.95075 |
| 23 | H9 | 2/3 | 1.42 | 0.145 | 1.06 | 0.815 |  |
| 24 | Layer 5 | 1 | 1.16 | 0.12 | 1.01 | 0.86 |  |
| 25 | Layer 5 | 1 | 1.03 | 0.146 | 0.868 | 0.494 |  |
| 26 | Layer 5 | 1 | 0.94 | 0.101 | 0.895 | 0.326 |  |
| 27 | Layer 5 | 1 | 0.903 | 0.0845 | 0.901 | 0.458 |  |
| 28 | Layer 5 | 1 | 0.955 | 0.115 | 0.94 | 0.49 | 30.8504 |
| **DAYINGZHUANG**  ***Chenopodium* sp** | | **AVERAGE** | **1.12** | **0.13** | **1.03** | **0.55** | **32.79** |
|  |  | **STDEV** | **0.15** | **0.04** | **0.14** | **0.15** | **5.14** |
