# Supplementary material for: Two-season agriculture and irrigated rice during the Dian: radiocarbon dates and archaeobotanical remains from Dayingzhuang, Yunnan, Southwest China
Source: Archaeol Anthropol Sci. 2021 Mar 13;13(4):62. doi: 10.1007/s12520-020-01268-y (PMC7956011; doi:10.1007/s12520-020-01268-y)
Supplement: Supplementary file 4 — (DOCX 83 kb) [file 12520_2020_1268_MOESM4_ESM.docx]

| Phytolith slide number | Samples ID | Sieved soil weight g | Dry pot weight g | Dry pot + phytolith weight g | Mounted phytolith weight mg |
| --- | --- | --- | --- | --- | --- |
| 1 | **34** | 1.26 | 6.82003 | 7.10618 | 2.39 |
| 2 | **32** | 1.26 | 6.78933 | 6.79419 | 2.41 |
| 3 | **30** | 1.32 | 6.29828 | 6.30318 | 2.32 |
| 4 | **26** | 1.34 | 6.82545 | 6.95833 | 2.84 |
| 5 | **24** | 1.19 | 6.69667 | 6.87281 | 2.48 |
| 6 | **22** | 1.25 | 6.91875 | 6.94076 | 2.39 |
| 7 | **20** | 1.27 | 6.971596 | 6.97956 | 2.49 |
| 8 | **18** | 1.29 | 6.92578 | 6.94179 | 2.44 |
| 9 | **16** | 1.21 | 6.78533 | 6.94351 | 2.32 |
| 11 | **10** | 1.55 | 6.72102 | 6.81199 | 2.42 |
| 12 | **3** | 1.49 | 6.80252 | 6.81044 | 2.37 |

| Sample ID | 3 | 10 | 16 | 18 | 20 | 22 |
| --- | --- | --- | --- | --- | --- | --- |
| SINGLE-CELL |  |  |  |  |  |  |
| SC rows counted | **21** | **2** | **2.5** | **3.5** | **2.5** | **3** |
| Elongate (Smooth): | 13 | 122 | 103 | 23 | 69 | 24 |
| Elongate (Sinuate) | 1 | 29 | 3 | 6 | 1 | 1 |
| Elongate (Echinate) | 7 | 23 | 19 | 42 | 39 | 32 |
| Elongate (Dendritic): |  | 25 | 23 | 9 | 3 | 26 |
| Elongate (Rods) |  |  | 3 |  |  |  |
| Stomata |  | 4 |  |  | 1 |  |
| Hair - long |  |  | 7 | 2 | 1 |  |
| Hair - segmented |  | 4 |  |  |  |  |
| Hair - acicular | 1 | 5 | 1 |  |  |  |
| Hair - uncifrom |  | 1 |  | 1 |  | 1 |
| Hair base |  |  |  |  |  |  |
| Bulliform: | 8 | 63 | 17 | 9 | 11 | 8 |
| Cuneiform bulliform | 1 | 15 |  | 21 | 7 | 8 |
| Oryza-type cuneiform bulliform |  | 7 |  | 3 | 18 | 2 |
| Oryza-type double peaked cell |  | 6 |  |  |  |  |
| Crenates: |  | 38 |  |  |  |  |
| Polylobate |  | 4 | 1 |  |  |  |
| Bilobes: | 3 | 4 | 17 | 32 | 18 | 44 |
| Setaria-type bilobe |  | 6 | 1 | 12 | 9 | 4 |
| Oryza-type bilobe |  | 19 | 1 | 7 | 4 | 4 |
| 1/2 bilobe |  | 2 | 1 |  |  | 1 |
| Cross - mirror image type |  | 17 |  |  | 2 | 4 |
| Cross - Bambusoidaea type |  |  | 3 | 1 |  | 1 |
| Cross - other | 4 | 10 |  | 8 | 1 | 11 |
| Rondels: | 12 | 1 | 43 | 54 | 38 | 40 |
| Stipa-Type Rondel | 2 | 2 |  | 4 | 6 | 3 |
| Saddles: | 3 |  | 20 | 36 | 28 | 43 |
| Collapsed saddle |  | 3 | 4 | 2 | 5 | 6 |
| Trapeziform short cell | 7 |  |  | 1 | 2 | 3 |
| Papillae | 4 | 1 | 5 |  |  | 2 |
| Sedge achene cell |  | 2 | 3 | 4 | 10 | 4 |
| Hat shaped |  | 4 | 5 | 5 | 5 | 8 |
| Reniform |  | 2 |  |  |  |  |
| Scutiform |  |  |  |  |  |  |
| Oval |  | 1 | 1 |  |  | 2 |
| Oblong | 1 |  |  |  |  | 1 |
| Conical | 1 | 1 | 4 | 6 |  | 4 |
| Conical with pointed apex | 1 |  |  |  | 3 | 2 |
| Globular smooth |  |  |  |  | 3 | 6 |
| Globular echinate |  |  |  |  |  |  |
| Golbular rugulose |  | 3 |  |  |  | 1 |
| Pitted sheet | 2 |  |  | 15 | 11 | 2 |
| Reticulate sheet |  |  |  |  |  |  |
| Sheet - other | 20 | 60 | 17 | 19 | 33 | 16 |
| Scalloped - cucurbitaceae |  |  |  | 2 |  |  |
| Scalloped - other |  | 5 |  | 4 | 2 |  |
| Tabluar |  | 2 |  |  |  |  |
| Trapeziform sinuate |  | 2 |  | 1 |  |  |
| Elongate - dicot/wood |  | 1 |  |  |  |  |
| Sample ID | **3** | **10** | **16** | **18** | **20** | **22** |
| Tracheids |  | 1 | 3 | 4 |  |  |
| Sclereid |  |  |  |  |  |  |
| Irregular block | 28 | 2 | 44 | 16 | 10 | 15 |
| Jigsaw puzzle (stellate) |  |  |  | 14 | 8 | 4 |
| Irregular (other) | 35 |  |  |  |  |  |
| MULTI-CELL |  |  |  |  |  |  |
| MC rows counted | **4** | **4** | **4** | **7** | **2** | **7** |
| Leaf/culm indet. - Palisade layer |  | 1 | 7 | 8 | 4 | 12 |
| Leaf/culm rondels |  |  |  |  |  |  |
| Leaf/culm bilobes | 1 |  | 1 |  |  | 2 |
| Leaf/culm saddles |  | 36 |  |  |  |  |
| Leaf/culm globular smooth |  |  |  |  |  |  |
| Leaf/culm globular rugulose |  |  |  |  |  |  |
| Leaf/culm globular echinate |  |  |  |  |  |  |
| Leaf/culm cross |  | 2 | 1 |  |  |  |
| Leaf/culm sedge |  |  |  |  |  |  |
| Leaf/culm square cell |  |  |  | 1 |  | 1 |
| Leaf/culm Oryza |  |  |  |  |  |  |
| Leaf/culm Setaria |  |  |  |  |  |  |
| Leaf jigsaw - upper epidermis |  |  | 2 |  |  | 1 |
| Awn |  |  | 1 |  |  |  |
| Unident husk | 8 |  | 40 | 45 | 34 | 34 |
| Indet dendritic husk |  |  |  |  |  |  |
| Oryza husk |  |  | 31 | 45 | 64 | 45 |
| Oryza double peaked cells | 1 | 3 | 5 | 7 |  | 1 |
| Oryza single peak cell |  |  | 5 | 3 | 9 | 4 |
| Wheat Husk |  |  | 4 | 6 | 1 | 8 |
| Barley Husk |  | 2 |  |  |  | 1 |
| Setaria-Type Husk |  |  | 3 | 6 |  | 2 |
| Panicum-Type husk |  |  |  |  |  |  |
| Millet type 1 - rounded processes |  | 3 |  |  |  |  |
| Millet type 2 - square processes |  |  |  |  |  |  |
| Cyperaceae (cones) |  | 1 |  |  |  |  |
| Polyhedral hair base |  |  | 1 |  |  |  |
| Verrucate |  | 1 |  |  |  |  |
| Scrobiculate |  |  |  |  |  |  |
| Rugulate |  |  |  |  |  |  |
| Striate |  | 7 | 1 |  |  |  |
| Tabular |  |  |  |  |  |  |
| Mesophyll - spongy layer |  |  | 1 |  |  |  |
| Indet multicell |  |  | 1 |  |  |  |
| Indet dicot |  |  | 2 | 1 |  | 3 |
| Silica aggregate |  |  |  | 2 | 5 | 6 |
| Diatoms | 4 |  | 5 | 1 | 1 | 5 |
| Starch |  | 5 | 2 |  |  |  |
| Sponge spicules |  | 3 |  |  |  |  |
| Indet silica forms | 2 | 1 |  |  |  |  |
| Parenchyma |  |  |  | 1 |  |  |
|  |  |  |  |  |  |  |

| Sample ID | 24 | 26 | 30 | 32 | 34 |
| --- | --- | --- | --- | --- | --- |
| SINGLE-CELL |  |  |  |  |  |
| SC rows counted | **9** | **21** | **22** | **16** | **5.5** |
| Elongate (Smooth): | 31 | 26 | 36 | 27 | 48 |
| Elongate (Sinuate) | 3 | 1 |  | 1 | 1 |
| Elongate (Echinate) | 47 | 41 | 33 | 26 | 39 |
| Elongate (Dendritic): | 17 | 45 | 1 | 1 | 7 |
| Elongate (Rods) |  |  |  |  |  |
| Stomata | 3 | 3 | 1 |  | 1 |
| Hair - long | 1 | 1 |  |  |  |
| Hair - segmented | 1 |  | 1 |  |  |
| Hair - acicular | 1 |  |  |  |  |
| Hair - uncifrom | 1 |  |  |  |  |
| Hair base |  |  |  |  | 2 |
| Bulliform: | 5 |  | 7 | 11 | 12 |
| Cuneiform bulliform | 26 | 7 | 22 | 9 | 12 |
| Oryza-type cuneiform bulliform | 3 | 3 | 6 | 3 | 7 |
| Oryza-type double peaked cell |  |  |  |  |  |
| Crenates: |  |  |  |  |  |
| Polylobate |  |  |  |  |  |
| Bilobes: | 30 | 22 | 11 | 12 | 7 |
| Setaria-type bilobe | 2 | 2 | 2 | 2 |  |
| Oryza-type bilobe | 5 | 4 |  |  |  |
| 1/2 bilobe |  |  |  |  |  |
| Cross - mirror image type | 6 | 4 | 3 |  |  |
| Cross - Bambusoidaea type |  |  |  |  |  |
| Cross - other |  | 6 |  |  | 7 |
| Rondels: | 54 | 39 | 38 | 37 | 45 |
| Stipa-Type Rondel |  |  |  |  |  |
| Saddles: | 35 | 31 | 21 | 22 | 37 |
| Collapsed saddle |  | 2 |  |  |  |
| Trapeziform short cell |  | 1 | 1 |  | 3 |
| Papillae |  |  |  |  |  |
| Sedge achene cell | 11 | 20 | 8 | 3 | 6 |
| Hat shaped | 13 | 10 | 2 |  |  |
| Reniform |  |  |  |  |  |
| Scutiform |  |  |  |  |  |
| Oval |  |  |  |  | 6 |
| Oblong |  |  |  |  |  |
| Conical |  | 5 | 5 | 1 | 14 |
| Conical with pointed apex |  |  | 4 | 3 | 1 |
| Globular smooth | 1 |  | 3 | 3 | 2 |
| Globular echinate |  |  |  |  |  |
| Golbular rugulose |  |  |  |  |  |
| Pitted sheet | 2 | 1 | 2 |  | 1 |
| Reticulate sheet |  |  |  |  |  |
| Sheet - other | 21 | 22 | 29 | 7 | 25 |
| Scalloped - cucurbitaceae |  |  |  |  |  |
| Scalloped - other | 3 |  |  |  | 1 |
| Tabluar |  |  |  |  |  |
| Trapeziform sinuate |  |  |  |  |  |
| Sample ID | **24** | **26** | **30** | **32** | **34** |
| Elongate - dicot/wood |  |  |  |  |  |
| Tracheids |  | 3 |  |  | 1 |
| Sclereid |  |  |  |  |  |
| Irregular block | 6 | 11 | 5 | 4 | 4 |
| Jigsaw puzzle (stellate) | 2 | 10 | 4 | 2 | 1 |
| Irregular (other) |  |  |  |  |  |
| MULTI-CELL |  |  |  |  |  |
| MC rows counted | **12** | **25** | **22** | **16** | **18** |
| Leaf/culm indet. - Palisade layer |  | 13 | 3 | 3 | 4 |
| Leaf/culm rondels |  |  |  |  |  |
| Leaf/culm bilobes |  |  |  |  |  |
| Leaf/culm saddles |  |  |  |  |  |
| Leaf/culm globular smooth |  |  |  | 3 |  |
| Leaf/culm globular rugulose |  |  |  |  |  |
| Leaf/culm globular echinate |  |  |  |  |  |
| Leaf/culm cross |  |  |  |  |  |
| Leaf/culm sedge |  |  |  |  |  |
| Leaf/culm square cell |  |  | 3 | 1 | 5 |
| Leaf/culm Oryza |  |  |  |  |  |
| Leaf/culm Setaria |  |  |  |  |  |
| Leaf jigsaw - upper epidermis |  |  |  |  |  |
| Awn |  |  |  |  |  |
| Unident husk |  | 8 | 6 | 4 | 29 |
| Indet dendritic husk | 1 |  |  |  |  |
| Oryza husk | 33 | 28 | 6 | 5 | 17 |
| Oryza double peaked cells | 31 | 6 | 3 | 2 |  |
| Oryza single peak cell | 35 | 3 | 11 | 4 | 6 |
| Wheat Husk |  | 2 |  | 1 |  |
| Barley Husk |  | 1 |  |  |  |
| Setaria-Type Husk | 1 |  |  |  |  |
| Panicum-Type husk |  |  |  |  |  |
| Millet type 1 - rounded processes | 2 | 4 |  |  | 1 |
| Millet type 2 - square processes |  |  |  |  |  |
| Cyperaceae (cones) |  |  |  |  |  |
| Polyhedral hair base |  |  |  |  |  |
| Verrucate |  |  |  |  |  |
| Scrobiculate |  |  |  |  |  |
| Rugulate |  |  |  |  |  |
| Striate |  |  |  |  |  |
| Tabular |  |  |  |  |  |
| Mesophyll - spongy layer |  |  |  |  |  |
| Indet multicell |  |  |  |  |  |
| Indet dicot |  | 1 | 1 | 2 | 1 |
| Silica aggregate |  | 2 | 1 |  | 1 |
| Diatoms |  | 6 | 5 |  | 3 |
| Starch |  |  |  |  |  |
| Sponge spicules |  |  |  |  |  |
| Indet silica forms |  |  |  |  |  |
| Parenchyma |  |  |  |  |  |

| Sample: | 3 | 10 | 16 | 18 | 20 | 22 |
| --- | --- | --- | --- | --- | --- | --- |
| SINGLE-CELL |  |  |  |  |  |  |
| Elongate (Smooth): | 67 | 71010 | 111434 | 1604 | 3336 | 2829 |
| Elongate (Sinuate) | 5 | 16879 | 3246 | 419 | 48 | 118 |
| Elongate (Echinate) | 36 | 13387 | 20556 | 2930 | 1886 | 3772 |
| Elongate (Dendritic): | 0 | 14551 | 28990 | 628 | 145 | 3065 |
| Elongate (Rods) | 0 | 0 | 129826 | 0 | 0 | 0 |
| Stomata | 0 | 2328 | 0 | 0 | 48 | 0 |
| Hair - long | 0 | 0 | 7573 | 140 | 48 | 0 |
| Hair - segmented | 0 | 2328 | 0 | 0 | 0 | 0 |
| Hair - acicular | 5 | 2910 | 1082 | 0 | 0 | 0 |
| Hair - uncifrom | 0 | 582 | 0 | 70 | 0 | 118 |
| Hair base | 0 | 0 | 0 | 0 | 0 | 0 |
| Bulliform: | 41 | 36669 | 18392 | 628 | 532 | 943 |
| Cuneiform bulliform | 5 | 8731 | 0 | 1465 | 338 | 943 |
| Oryza-type cuneiform bulliform | 0 | 4074 | 0 | 209 | 870 | 236 |
| Oryza-type double peaked cell | 0 | 3492 | 0 | 0 | 0 | 0 |
| Crenates: | 0 | 22118 | 0 | 0 | 0 | 0 |
| Polylobate | 0 | 2328 | 1082 | 0 | 0 | 0 |
| Bilobes: | 15 | 2328 | 18392 | 2232 | 870 | 5187 |
| Setaria-type bilobe | 0 | 3492 | 1082 | 837 | 435 | 472 |
| Oryza-type bilobe | 0 | 11059 | 1082 | 488 | 193 | 472 |
| 1/2 bilobe | 0 | 1164 | 1082 | 0 | 0 | 118 |
| Cross - mirror image type | 0 | 9895 | 0 | 0 | 97 | 472 |
| Cross - Bambusoidaea type | 0 | 0 | 3246 | 70 | 0 | 118 |
| Cross - other | 21 | 5820 | 0 | 558 | 48 | 1297 |
| Rondels: | 62 | 582 | 46521 | 3767 | 1837 | 4715 |
| Stipa-Type Rondel | 10 | 1164 | 0 | 279 | 290 | 354 |
| Saddles: | 15 | 0 | 21638 | 2511 | 1354 | 5069 |
| Collapsed saddle | 0 | 1746 | 4328 | 140 | 242 | 707 |
| Trapeziform short cell | 36 | 0 | 0 | 70 | 97 | 354 |
| Papillae | 21 | 582 | 5409 | 0 | 0 | 236 |
| Sedge achene cell | 0 | 1164 | 3246 | 279 | 484 | 472 |
|  | 0 | 2328 | 5409 | 349 | 242 | 943 |
| Reniform | 0 | 1164 | 0 | 0 | 0 | 0 |
| Scutiform | 0 | 0 | 0 | 0 | 0 | 0 |
| Oval | 0 | 582 | 1082 | 0 | 0 | 236 |
| Oblong | 5 | 0 | 0 | 0 | 0 | 118 |
| Conical | 5 | 582 | 4328 | 419 | 0 | 472 |
| Conical with pointed apex | 5 | 0 | 0 | 0 | 145 | 236 |
| Globular smooth | 0 | 0 | 0 | 0 | 145 | 707 |
| Globular echinate | 0 | 0 | 0 | 0 | 0 | 0 |
| Golbular rugulose | 0 | 1746 | 0 | 0 | 0 | 118 |
| Pitted sheet | 10 | 0 | 0 | 1046 | 532 | 236 |
| Reticulate sheet | 0 | 0 | 0 | 0 | 0 | 0 |
| Sheet - other | 103 | 34923 | 18392 | 1325 | 1596 | 1886 |
| Scalloped - cucurbitaceae | 0 | 0 | 0 | 140 | 0 | 0 |
| Scalloped - other | 0 | 2910 | 0 | 279 | 97 | 0 |
| Tabluar | 0 | 1164 | 0 | 0 | 0 | 0 |
| Trapeziform sinuate | 0 | 1164 | 0 | 70 | 0 | 0 |
| Sample: | **3** | **10** | **16** | **18** | **20** | **22** |
| Elongate - dicot/wood | 0 | 582 | 0 | 0 | 0 | 0 |
| Tracheids | 0 | 582 | 3246 | 279 | 0 | 0 |
| Sclereid | 0 | 0 | 0 | 0 | 0 | 0 |
| Irregular block | 144 | 1164 | 47603 | 1116 | 484 | 1768 |
| Jigsaw puzzle (stellate) | 0 | 0 | 0 | 977 | 387 | 472 |
| Irregular (other) | 179 | 0 | 0 | 0 | 0 | 0 |
| MULTI-CELL |  |  |  |  |  |  |
| Leaf/culm indet. - Palisade layer | 0 | 291 | 113597 | 13393 | 3316 | 606 |
| Leaf/culm rondels | 0 | 0 | 0 | 0 | 0 | 0 |
| Leaf/culm bilobes | 27 | 0 | 16228 | 0 | 0 | 101 |
| Leaf/culm saddles | 0 | 10477 | 0 | 0 | 0 | 0 |
| Leaf/culm globular smooth | 0 | 0 | 0 | 0 | 0 | 0 |
| Leaf/culm globular rugulose | 0 | 0 | 0 | 0 | 0 | 0 |
| Leaf/culm globular echinate | 0 | 0 | 0 | 0 | 0 | 0 |
| Leaf/culm cross | 0 | 582 | 16228 | 0 | 0 | 0 |
| Leaf/culm sedge | 0 | 0 | 0 | 0 | 0 | 0 |
| Leaf/culm square cell | 0 | 0 | 0 | 1674 | 0 | 51 |
| Leaf/culm Oryza | 0 | 0 | 0 | 0 | 0 | 0 |
| Leaf/culm Setaria | 0 | 0 | 0 | 0 | 0 | 0 |
| Leaf jigsaw - upper epidermis | 0 | 0 | 32456 | 0 | 0 | 51 |
| Awn | 0 | 0 | 16228 | 0 | 0 | 0 |
| Unident husk | 215 | 0 | 649129 | 75337 | 28183 | 1718 |
| Unindet dendritic husk | 0 | 0 | 0 | 0 | 0 | 0 |
| Oryza husk | 0 | 0 | 503075 | 75337 | 53051 | 2273 |
| Oryza double peaked cells | 27 | 873 | 81141 | 11719 | 0 | 51 |
| Oryza single peak | 0 | 0 | 81141 | 5022 | 7460 | 202 |
| Wheat Husk | 0 | 0 | 64913 | 10045 | 829 | 404 |
| Barley Husk | 0 | 582 | 0 | 0 | 0 | 51 |
| Setaria-Type Husk | 0 | 0 | 48685 | 10045 | 0 | 101 |
| Panicum-Type husk | 0 | 0 | 0 | 0 | 0 | 0 |
| Millet type 1 - rounded processes | 0 | 873 | 0 | 0 | 0 | 0 |
| Millet type 2 - square processes | 0 | 0 | 0 | 0 | 0 | 0 |
| Cyperaceae (cones) | 0 | 291 | 0 | 0 | 0 | 0 |
| Polyhedral hair base | 0 | 0 | 16228 | 0 | 0 | 0 |
| Verrucate | 0 | 291 | 0 | 0 | 0 | 0 |
| Scrobiculate | 0 | 0 | 0 | 0 | 0 | 0 |
| Rugulate | 0 | 0 | 0 | 0 | 0 | 0 |
| Striate | 0 | 2037 | 16228 | 0 | 0 | 0 |
| Tabular | 0 | 0 | 0 | 0 | 0 | 0 |
| Mesophyll - spongy layer | 0 | 0 | 16228 | 0 | 0 | 0 |
| Indet multicell | 0 | 0 | 16228 | 0 | 0 | 0 |
| Indet dicot | 0 | 0 | 32456 | 1674 | 0 | 152 |
| Silica aggregate | 0 | 0 | 0 | 3348 | 4145 | 303 |
| Diatoms | 108 | 0 | 81141 | 1674 | 829 | 253 |
| Starch | 0 | 1455 | 32456 | 0 | 0 | 0 |
| Sponge spicules | 0 | 873 | 0 | 0 | 0 | 0 |
| Indet silica forms | 54 | 291 | 0 | 0 | 0 | 0 |
| Sample: | **3** | **10** | **16** | **18** | **20** | **22** |
| Parenchyma | 0 | 0 | 0 | 1674 | 0 | 0 |
| Total % phytoliths per gram | 0.531476510067114 | 5.86896774193548 | 13.0727272727273 | 0.627086614173228 | 0.627086614173228 | 1.760792 |

| Sample: | 24 | 26 | 30 | 32 | 34 |
| --- | --- | --- | --- | --- | --- |
| SINGLE-CELL |  |  |  |  |  |
| Elongate (Smooth): | 9869 | 2075 | 126 | 130 | 39806 |
| Elongate (Sinuate) | 955 | 80 | 0 | 5 | 829 |
| Elongate (Echinate) | 14963 | 3272 | 115 | 125 | 32342 |
| Elongate (Dendritic): | 5412 | 3591 | 3 | 5 | 5805 |
| Elongate (Rods) | 0 | 0 | 0 | 0 | 0 |
| Stomata | 955 | 239 | 3 | 0 | 829 |
| Hair - long | 318 | 80 | 0 | 0 | 0 |
| Hair - segmented | 318 | 0 | 3 | 0 | 0 |
| Hair - acicular | 318 | 0 | 0 | 0 | 0 |
| Hair - uncifrom | 318 | 0 | 0 | 0 | 0 |
| Hair base | 0 | 0 | 0 | 0 | 1659 |
| Bulliform: | 1592 | 0 | 24 | 53 | 9951 |
| Cuneiform bulliform | 8278 | 559 | 77 | 43 | 9951 |
| Oryza-type cuneiform bulliform | 955 | 239 | 21 | 14 | 5805 |
| Oryza-type double peaked cell | 0 | 0 | 0 | 0 | 0 |
| Crenates: | 0 | 0 | 0 | 0 | 0 |
| Polylobate | 0 | 0 | 0 | 0 | 0 |
| Bilobes: | 9551 | 1756 | 38 | 58 | 5805 |
| Setaria-type bilobe | 637 | 160 | 7 | 10 | 0 |
| Oryza-type bilobe | 1592 | 319 | 0 | 0 | 0 |
| 1/2 bilobe | 0 | 0 | 0 | 0 | 0 |
| Cross - mirror image type | 1910 | 319 | 10 | 0 | 0 |
| Cross - Bambusoidaea type | 0 | 0 | 0 | 0 | 0 |
| Cross - other | 0 | 479 | 0 | 0 | 5805 |
| Rondels: | 17192 | 3113 | 133 | 178 | 37318 |
| Stipa-Type Rondel | 0 | 0 | 0 | 0 | 0 |
| Saddles: | 11143 | 2474 | 73 | 106 | 30684 |
| Collapsed saddle | 0 | 160 | 0 | 0 | 0 |
| Trapeziform short cell | 0 | 80 | 3 | 0 | 2488 |
| Papillae | 0 | 0 | 0 | 0 | 0 |
| Sedge achene cell | 3502 | 1596 | 28 | 14 | 4976 |
| Hat shaped | 4139 | 798 | 7 | 0 | 0 |
| Reniform | 0 | 0 | 0 | 0 | 0 |
| Scutiform | 0 | 0 | 0 | 0 | 0 |
| Oval | 0 | 0 | 0 | 0 | 4976 |
| Oblong | 0 | 0 | 0 | 0 | 0 |
| Conical | 0 | 399 | 17 | 5 | 11610 |
| Conical with pointed apex | 0 | 0 | 14 | 14 | 829 |
| Globular smooth | 318 | 0 | 10 | 14 | 1659 |
| Globular echinate | 0 | 0 | 0 | 0 | 0 |
| Sample: | **24** | **26** | **30** | **32** | **34** |
| Globular rugulose | 0 | 0 | 0 | 0 | 0 |
| Pitted sheet | 637 | 80 | 7 | 0 | 829 |
| Reticulate sheet | 0 | 0 | 0 | 0 | 0 |
| Sheet - other | 6686 | 1756 | 101 | 34 | 20732 |
| Scalloped - cucurbitaceae | 0 | 0 | 0 | 0 | 0 |
| Scalloped - other | 955 | 0 | 0 | 0 | 829 |
| Tabluar | 0 | 0 | 0 | 0 | 0 |
| Trapeziform sinuate | 0 | 0 | 0 | 0 | 0 |
| Elongate - dicot/wood | 0 | 0 | 0 | 0 | 0 |
| Tracheids | 0 | 239 | 0 | 0 | 829 |
| Sclereid | 0 | 0 | 0 | 0 | 0 |
| Irregular block | 1910 | 878 | 17 | 19 | 3317 |
| Jigsaw puzzle (stellate) | 637 | 798 | 14 | 10 | 829 |
| Irregular (other) | 0 | 0 | 0 | 0 | 0 |
| MULTI-CELL |  |  |  |  |  |
| Leaf/culm indet. - Palisade layer | 0 | 872 | 10 | 14 | 1014 |
| Leaf/culm rondels | 0 | 0 | 0 | 0 | 0 |
| Leaf/culm bilobes | 0 | 0 | 0 | 0 | 0 |
| Leaf/culm saddles | 0 | 0 | 0 | 0 | 0 |
| Leaf/culm globular smooth | 0 | 0 | 0 | 14 | 0 |
| Leaf/culm globular rugulose | 0 | 0 | 0 | 0 | 0 |
| Leaf/culm globular echinate | 0 | 0 | 0 | 0 | 0 |
| Leaf/culm cross | 0 | 0 | 0 | 0 | 0 |
| Leaf/culm sedge | 0 | 0 | 0 | 0 | 0 |
| Leaf/culm square cell | 0 | 0 | 10 | 5 | 1267 |
| Leaf/culm Oryza | 0 | 0 | 0 | 0 | 0 |
| Leaf/culm Setaria | 0 | 0 | 0 | 0 | 0 |
| Leaf jigsaw - upper epidermis | 0 | 0 | 0 | 0 | 0 |
| Awn | 0 | 0 | 0 | 0 | 0 |
| Unident husk | 0 | 536 | 21 | 19 | 7348 |
| Unindet dendritic husk | 239 | 0 | 0 | 0 | 0 |
| Oryza husk | 7880 | 1877 | 21 | 24 | 4308 |
| Oryza double peaked cells | 7402 | 402 | 10 | 10 | 0 |
| Oryza single peak | 8357 | 201 | 38 | 19 | 1520 |
| Wheat Husk | 0 | 134 | 0 | 5 | 0 |
| Barley Husk | 0 | 67 | 0 | 0 | 0 |
| Setaria-Type Husk | 239 | 0 | 0 | 0 | 0 |
| Panicum-Type husk | 0 | 0 | 0 | 0 | 0 |
| Millet type 1 - rounded processes | 478 | 268 | 0 | 0 | 253 |
| Millet type 2 - square processes | 0 | 0 | 0 | 0 | 0 |
| Cyperaceae (cones) | 0 | 0 | 0 | 0 | 0 |
| Polyhedral hair base | 0 | 0 | 0 | 0 | 0 |
| Verrucate | 0 | 0 | 0 | 0 | 0 |
| Scrobiculate | 0 | 0 | 0 | 0 | 0 |
| Rugulate | 0 | 0 | 0 | 0 | 0 |
| Striate | 0 | 0 | 0 | 0 | 0 |
| Sample: | **24** | **26** | **30** | **32** | **34** |
| Tabular | 0 | 0 | 0 | 0 | 0 |
| Mesophyll - spongy layer | 0 | 0 | 0 | 0 | 0 |
| Indet multicell | 0 | 0 | 0 | 0 | 0 |
| Indet dicot | 0 | 67 | 3 | 10 | 253 |
| Silica aggregate | 0 | 134 | 3 | 0 | 253 |
| Diatoms | 0 | 402 | 17 | 0 | 760 |
| Starch | 0 | 0 | 0 | 0 | 0 |
| Sponge spicules | 0 | 0 | 0 | 0 | 0 |
| Indet silica forms | 0 | 0 | 0 | 0 | 0 |
| Parenchyma | 0 | 0 | 0 | 0 | 0 |
| Total % phytoliths per gram | 14.8042016806723 | 9.91641791044776 | 0.371212121212121 | 0.385714285714286 | 22.7103174603175 |

| Sample: | 34 | 32 | 30 | 26 | 24 |
| --- | --- | --- | --- | --- | --- |
| Layer | **5** | **5** | **5** | **5** | **4** |
| SINGLE-CELL |  |  |  |  |  |
| Elongate (Smooth): | 15.46 | 13.57 | 12.68 | 6.80 | 7.61 |
| Elongate (Sinuate) | 0.32 | 0.50 | 0.00 | 0.26 | 0.74 |
| Elongate (Echinate) | 12.56 | 13.07 | 11.62 | 10.73 | 11.54 |
| Elongate (Dendritic): | 2.25 | 0.50 | 0.35 | 11.78 | 4.17 |
| Elongate (Rods) | 0.00 | 0.00 | 0.00 | 0.00 | 0.00 |
| Stomata | 0.32 | 0.00 | 0.35 | 0.79 | 0.74 |
| Hair - long | 0.00 | 0.00 | 0.00 | 0.26 | 0.25 |
| Hair - segmented | 0.00 | 0.00 | 0.35 | 0.00 | 0.25 |
| Hair - acicular | 0.00 | 0.00 | 0.00 | 0.00 | 0.25 |
| Hair - uncifrom | 0.00 | 0.00 | 0.00 | 0.00 | 0.25 |
| Hair base | 0.64 | 0.00 | 0.00 | 0.00 | 0.00 |
| Bulliform: | 3.87 | 5.53 | 2.46 | 0.00 | 1.23 |
| Cuneiform bulliform | 3.87 | 4.52 | 7.75 | 1.83 | 6.38 |
| Oryza-type cuneiform bulliform | 2.25 | 1.51 | 2.11 | 0.79 | 0.74 |
| Oryza-type double peaked cell | 0.00 | 0.00 | 0.00 | 0.00 | 0.00 |
| Crenates: | 0.00 | 0.00 | 0.00 | 0.00 | 0.00 |
| Polylobate | 0.00 | 0.00 | 0.00 | 0.00 | 0.00 |
| Bilobes: | 2.25 | 6.03 | 3.87 | 5.76 | 7.37 |
| Setaria-type bilobe | 0.00 | 1.01 | 0.70 | 0.52 | 0.49 |
| Oryza-type bilobe | 0.00 | 0.00 | 0.00 | 1.05 | 1.23 |
| 1/2 bilobe | 0.00 | 0.00 | 0.00 | 0.00 | 0.00 |
| Cross - mirror image type | 0.00 | 0.00 | 1.06 | 1.05 | 1.47 |
| Cross - Bambusoidaea type | 0.00 | 0.00 | 0.00 | 0.00 | 0.00 |
| Cross - other | 2.25 | 0.00 | 0.00 | 1.57 | 0.00 |
| Rondels: | 14.49 | 18.59 | 13.38 | 10.21 | 13.26 |
| Stipa-Type Rondel | 0.00 | 0.00 | 0.00 | 0.00 | 0.00 |
| Saddles: | 11.92 | 11.06 | 7.39 | 8.11 | 8.59 |
| Collapsed saddle | 0.00 | 0.00 | 0.00 | 0.52 | 0.00 |
| Trapeziform short cell | 0.97 | 0.00 | 0.35 | 0.26 | 0.00 |
| Papillae | 0.00 | 0.00 | 0.00 | 0.00 | 0.00 |
| Sedge achene cell | 1.93 | 1.51 | 2.82 | 5.23 | 2.70 |
| Hat shaped | 0.00 | 0.00 | 0.70 | 2.62 | 3.19 |
| Reniform | 0.00 | 0.00 | 0.00 | 0.00 | 0.00 |
| Scutiform | 0.00 | 0.00 | 0.00 | 0.00 | 0.00 |
| Oval | 1.93 | 0.00 | 0.00 | 0.00 | 0.00 |
| Oblong | 0.00 | 0.00 | 0.00 | 0.00 | 0.00 |
| Conical | 4.51 | 0.50 | 1.76 | 1.31 | 0.00 |
| Trichomes | 0.32 | 1.51 | 1.41 | 0.00 | 0.00 |
| Globular smooth | 0.64 | 1.51 | 1.06 | 0.00 | 0.25 |
| Golbular rugulose | 0.00 | 0.00 | 0.00 | 0.00 | 0.00 |
| Pitted sheet | 0.32 | 0.00 | 0.70 | 0.26 | 0.49 |
| Reticulate sheet | 0.00 | 0.00 | 0.00 | 0.00 | 0.00 |
| Sheet - other | 8.05 | 3.52 | 10.21 | 5.76 | 5.16 |
| Scalloped - cucurbitaceae | 0.00 | 0.00 | 0.00 | 0.00 | 0.00 |
| Scalloped - other | 0.32 | 0.00 | 0.00 | 0.00 | 0.74 |
| Tabluar | 0.00 | 0.00 | 0.00 | 0.00 | 0.00 |
| Trapeziform sinuate | 0.00 | 0.00 | 0.00 | 0.00 | 0.00 |
| Elongate - dicot/wood | 0.00 | 0.00 | 0.00 | 0.00 | 0.00 |
| Sample: | **34** | **32** | **30** | **26** | **24** |
| Tracheids | 0.32 | 0.00 | 0.00 | 0.79 | 0.00 |
| Irregular block | 1.29 | 2.01 | 1.76 | 2.88 | 1.47 |
| Jigsaw puzzle (stellate) | 0.32 | 1.01 | 1.41 | 2.62 | 0.49 |
| Irregular (other) | 0.00 | 0.00 | 0.00 | 0.00 | 0.00 |
| MULTI-CELL |  |  |  |  |  |
| Leaf/culm indet. - Palisade layer | 0.39 | 1.51 | 1.06 | 2.86 | 0.00 |
| Leaf/culm rondels | 0.00 | 0.00 | 0.00 | 0.00 | 0.00 |
| Leaf/culm bilobes | 0.00 | 0.00 | 0.00 | 0.00 | 0.00 |
| Leaf/culm saddles | 0.00 | 0.00 | 0.00 | 0.00 | 0.00 |
| Leaf/culm globular smooth | 0.00 | 1.51 | 0.00 | 0.00 | 0.00 |
| Leaf/culm globular rugulose | 0.00 | 0.00 | 0.00 | 0.00 | 0.00 |
| Leaf/culm globular echinate | 0.00 | 0.00 | 0.00 | 0.00 | 0.00 |
| Leaf/culm cross | 0.00 | 0.00 | 0.00 | 0.00 | 0.00 |
| Leaf/culm sedge | 0.00 | 0.00 | 0.00 | 0.00 | 0.00 |
| Leaf/culm square ? polyhedral | 0.49 | 0.50 | 1.06 | 0.00 | 0.00 |
| Leaf jigsaw - upper epidermis | 0.00 | 0.00 | 0.00 | 0.00 | 0.00 |
| Awn | 0.00 | 0.00 | 0.00 | 0.00 | 0.00 |
| Unident husk | 2.85 | 2.01 | 2.11 | 1.76 | 0.00 |
| Unindet dendritic husk | 0.00 | 0.00 | 0.00 | 0.00 | 0.18 |
| Oryza husk | 1.67 | 2.51 | 2.11 | 6.15 | 6.08 |
| Oryza double peaked cells | 0.00 | 1.01 | 1.06 | 1.32 | 5.71 |
| Oryza single peak | 0.59 | 2.01 | 3.87 | 0.66 | 6.45 |
| Wheat Husk | 0.00 | 0.50 | 0.00 | 0.44 | 0.00 |
| Barley Husk | 0.00 | 0.00 | 0.00 | 0.22 | 0.00 |
| Setaria-Type Husk | 0.00 | 0.00 | 0.00 | 0.00 | 0.18 |
| Millet type 1 - rounded processes | 0.10 | 0.00 | 0.00 | 0.88 | 0.37 |
| Millet type 2 - square processes | 0.00 | 0.00 | 0.00 | 0.00 | 0.00 |
| Cyperaceae (cones) | 0.00 | 0.00 | 0.00 | 0.00 | 0.00 |
| Polyhedral hair base | 0.00 | 0.00 | 0.00 | 0.00 | 0.00 |
| Verrucate | 0.00 | 0.00 | 0.00 | 0.00 | 0.00 |
| Striate | 0.00 | 0.00 | 0.00 | 0.00 | 0.00 |
| Mesophyll - spongy layer | 0.00 | 0.00 | 0.00 | 0.00 | 0.00 |
| Indet multicell | 0.00 | 0.00 | 0.00 | 0.00 | 0.00 |
| Indet dicot | 0.10 | 1.01 | 0.35 | 0.22 | 0.00 |
| Silica aggregate | 0.10 | 0.00 | 0.35 | 0.44 | 0.00 |
| Diatoms | 0.30 | 0.00 | 1.76 | 1.32 | 0.00 |
| Starch | 0.00 | 0.00 | 0.00 | 0.00 | 0.00 |
| Sponge spicules | 0.00 | 0.00 | 0.00 | 0.00 | 0.00 |
| Indet silica forms | 0.00 | 0.00 | 0.00 | 0.00 | 0.00 |
| Parenchyma | 0.00 | 0.00 | 0.00 | 0.00 | 0.00 |
| TOTAL | **100.00** | **100.00** | **100.00** | **100.00** | **100.00** |

| Sample: | 22 | 20 | 18 | 16 | 10 | 3 |
| --- | --- | --- | --- | --- | --- | --- |
| Layer | **4** | **4** | **4** | **3** | **modern** | **modern** |
| SINGLE-CELL |  |  |  |  |  |  |
| Elongate (Smooth): | 6.21 | 2.91 | 0.68 | 4.76 | 23.13 | 6.75 |
| Elongate (Sinuate) | 0.26 | 0.04 | 0.18 | 0.14 | 5.50 | 0.52 |
| Elongate (Echinate) | 8.28 | 1.64 | 1.25 | 0.88 | 4.36 | 3.64 |
| Elongate (Dendritic): | 6.73 | 0.13 | 0.27 | 1.24 | 4.74 | 0.00 |
| Elongate (Rods) | 0.00 | 0.00 | 0.00 | 5.54 | 0.00 | 0.00 |
| Stomata | 0.00 | 0.04 | 0.00 | 0.00 | 0.76 | 0.00 |
| Hair - long | 0.00 | 0.04 | 0.06 | 0.32 | 0.00 | 0.00 |
| Hair - segmented | 0.00 | 0.00 | 0.00 | 0.00 | 0.76 | 0.00 |
| Hair - acicular | 0.00 | 0.00 | 0.00 | 0.05 | 0.95 | 0.52 |
| Hair - uncifrom | 0.26 | 0.00 | 0.03 | 0.00 | 0.19 | 0.00 |
| Hair base | 0.00 | 0.00 | 0.00 | 0.00 | 0.00 | 0.00 |
| Bulliform: | 2.07 | 0.46 | 0.27 | 0.79 | 11.94 | 4.16 |
| Cuneiform bulliform | 2.07 | 0.30 | 0.62 | 0.00 | 2.84 | 0.52 |
| Oryza-type cuneiform bulliform | 0.52 | 0.76 | 0.09 | 0.00 | 1.33 | 0.00 |
| Oryza-type double peaked cell | 0.00 | 0.00 | 0.00 | 0.00 | 1.14 | 0.00 |
| Crenates: | 0.00 | 0.00 | 0.00 | 0.00 | 7.20 | 0.00 |
| Polylobate | 0.00 | 0.00 | 0.00 | 0.05 | 0.76 | 0.00 |
| Bilobes: | 11.38 | 0.76 | 0.95 | 0.79 | 0.76 | 1.56 |
| Setaria-type bilobe | 1.03 | 0.38 | 0.36 | 0.05 | 1.14 | 0.00 |
| Oryza-type bilobe | 1.03 | 0.17 | 0.21 | 0.05 | 3.60 | 0.00 |
| 1/2 bilobe | 0.26 | 0.00 | 0.00 | 0.05 | 0.38 | 0.00 |
| Cross - mirror image type | 1.03 | 0.08 | 0.00 | 0.00 | 3.22 | 0.00 |
| Cross - Bambusoidaea type | 0.26 | 0.00 | 0.03 | 0.14 | 0.00 | 0.00 |
| Cross - other | 2.85 | 0.04 | 0.24 | 0.00 | 1.90 | 2.08 |
| Rondels: | 10.35 | 1.60 | 1.61 | 1.99 | 0.19 | 6.23 |
| Stipa-Type Rondel | 0.78 | 0.25 | 0.12 | 0.00 | 0.38 | 1.04 |
| Saddles: | 11.12 | 1.18 | 1.07 | 0.92 | 0.00 | 1.56 |
| Collapsed saddle | 1.55 | 0.21 | 0.06 | 0.18 | 0.57 | 0.00 |
| Trapeziform short cell | 0.78 | 0.08 | 0.03 | 0.00 | 0.00 | 3.64 |
| Papillae | 0.52 | 0.00 | 0.00 | 0.23 | 0.19 | 2.08 |
| Sedge achene cell | 1.03 | 0.42 | 0.12 | 0.14 | 0.38 | 0.00 |
| Hat shaped | 2.07 | 0.21 | 0.15 | 0.23 | 0.76 | 0.00 |
| Reniform | 0.00 | 0.00 | 0.00 | 0.00 | 0.38 | 0.00 |
| Scutiform | 0.00 | 0.00 | 0.00 | 0.00 | 0.00 | 0.00 |
| Oval | 0.52 | 0.00 | 0.00 | 0.05 | 0.19 | 0.00 |
| Oblong | 0.26 | 0.00 | 0.00 | 0.00 | 0.00 | 0.52 |
| Conical | 1.03 | 0.00 | 0.18 | 0.18 | 0.19 | 0.52 |
| Trichomes | 0.52 | 0.13 | 0.00 | 0.00 | 0.00 | 0.52 |
| Globular smooth | 1.55 | 0.13 | 0.00 | 0.00 | 0.00 | 0.00 |
| Golbular rugulose | 0.26 | 0.00 | 0.00 | 0.00 | 0.57 | 0.00 |
| Pitted sheet | 0.52 | 0.46 | 0.45 | 0.00 | 0.00 | 1.04 |
| Reticulate sheet | 0.00 | 0.00 | 0.00 | 0.00 | 0.00 | 0.00 |
| Sheet - other | 4.14 | 1.39 | 0.56 | 0.79 | 11.37 | 10.39 |
| Scalloped - cucurbitaceae | 0.00 | 0.00 | 0.06 | 0.00 | 0.00 | 0.00 |
| Scalloped - other | 0.00 | 0.08 | 0.12 | 0.00 | 0.95 | 0.00 |
| Tabluar | 0.00 | 0.00 | 0.00 | 0.00 | 0.38 | 0.00 |
| Trapeziform sinuate | 0.00 | 0.00 | 0.03 | 0.00 | 0.38 | 0.00 |
| Elongate - dicot/wood | 0.00 | 0.00 | 0.00 | 0.00 | 0.19 | 0.00 |
| Tracheids | 0.00 | 0.00 | 0.12 | 0.14 | 0.19 | 0.00 |
| Sample: | **22** | **20** | **18** | **16** | **10** | **3** |
| Irregular block | 3.88 | 0.42 | 0.48 | 2.03 | 0.38 | 14.55 |
| Jigsaw puzzle (stellate) | 1.03 | 0.34 | 0.42 | 0.00 | 0.00 | 0.00 |
| Irregular (other) | 0.00 | 0.00 | 0.00 | 0.00 | 0.00 | 18.18 |
| MULTI-CELL |  |  |  |  |  |  |
| Leaf/culm indet. - Palisade layer | 1.33 | 2.89 | 5.71 | 4.85 | 0.09 | 0.00 |
| Leaf/culm rondels | 0.00 | 0.00 | 0.00 | 0.00 | 0.00 | 0.00 |
| Leaf/culm bilobes | 0.22 | 0.00 | 0.00 | 0.69 | 0.00 | 2.73 |
| Leaf/culm saddles | 0.00 | 0.00 | 0.00 | 0.00 | 3.41 | 0.00 |
| Leaf/culm globular smooth | 0.00 | 0.00 | 0.00 | 0.00 | 0.00 | 0.00 |
| Leaf/culm globular rugulose | 0.00 | 0.00 | 0.00 | 0.00 | 0.00 | 0.00 |
| Leaf/culm globular echinate | 0.00 | 0.00 | 0.00 | 0.00 | 0.00 | 0.00 |
| Leaf/culm cross | 0.00 | 0.00 | 0.00 | 0.69 | 0.19 | 0.00 |
| Leaf/culm sedge | 0.00 | 0.00 | 0.00 | 0.00 | 0.00 | 0.00 |
| Leaf/culm square ? polyhedral | 0.11 | 0.00 | 0.71 | 0.00 | 0.00 | 0.00 |
| Leaf jigsaw - upper epidermis | 0.11 | 0.00 | 0.00 | 1.39 | 0.00 | 0.00 |
| Awn | 0.00 | 0.00 | 0.00 | 0.69 | 0.00 | 0.00 |
| Unident husk | 3.77 | 24.58 | 32.11 | 27.72 | 0.00 | 21.82 |
| Unindet dendritic husk | 0.00 | 0.00 | 0.00 | 0.00 | 0.00 | 0.00 |
| Oryza husk | 4.99 | 46.28 | 32.11 | 21.48 | 0.00 | 0.00 |
| Oryza double peaked cells | 0.11 | 0.00 | 5.00 | 3.46 | 0.28 | 2.73 |
| Oryza single peak | 0.44 | 6.51 | 2.14 | 3.46 | 0.00 | 0.00 |
| Wheat Husk | 0.89 | 0.72 | 4.28 | 2.77 | 0.00 | 0.00 |
| Barley Husk | 0.11 | 0.00 | 0.00 | 0.00 | 0.19 | 0.00 |
| Setaria-Type Husk | 0.22 | 0.00 | 4.28 | 2.08 | 0.00 | 0.00 |
| Millet type 1 - rounded processes | 0.00 | 0.00 | 0.00 | 0.00 | 0.28 | 0.00 |
| Millet type 2 - square processes | 0.00 | 0.00 | 0.00 | 0.00 | 0.00 | 0.00 |
| Cyperaceae (cones) | 0.00 | 0.00 | 0.00 | 0.00 | 0.09 | 0.00 |
| Polyhedral hair base | 0.00 | 0.00 | 0.00 | 0.69 | 0.00 | 0.00 |
| Verrucate | 0.00 | 0.00 | 0.00 | 0.00 | 0.09 | 0.00 |
| Striate | 0.00 | 0.00 | 0.00 | 0.69 | 0.66 | 0.00 |
| Mesophyll - spongy layer | 0.00 | 0.00 | 0.00 | 0.69 | 0.00 | 0.00 |
| Indet multicell | 0.00 | 0.00 | 0.00 | 0.69 | 0.00 | 0.00 |
| Indet dicot | 0.33 | 0.00 | 0.71 | 1.39 | 0.00 | 0.00 |
| Silica aggregate | 0.67 | 3.62 | 1.43 | 0.00 | 0.00 | 0.00 |
| Diatoms | 0.55 | 0.72 | 0.71 | 3.46 | 0.00 | 10.91 |
| Starch | 0.00 | 0.00 | 0.00 | 1.39 | 0.47 | 0.00 |
| Sponge spicules | 0.00 | 0.00 | 0.00 | 0.00 | 0.28 | 0.00 |
| Indet silica forms | 0.00 | 0.00 | 0.00 | 0.00 | 0.09 | 5.45 |
| Parenchyma | 0.00 | 0.00 | 0.71 | 0.00 | 0.00 | 0.00 |
| TOTAL | **100.00** | **100.00** | **100.00** | **100.00** | **100.00** | **100.00** |
